# A Social Media–Based Support Group for Youth Living With HIV in Nigeria (SMART Connections): Randomized Controlled Trial

**DOI:** 10.2196/18343

**Published:** 2020-06-02

**Authors:** Lisa Dulli, Kathleen Ridgeway, Catherine Packer, Kate R Murray, Tolulope Mumuni, Kate F Plourde, Mario Chen, Adesola Olumide, Oladosu Ojengbede, Donna R McCarraher

**Affiliations:** 1 FHI 360 Durham, NC United States; 2 Center for Population and Reproductive Health College of Medicine University of Ibadan Ibadan Nigeria

**Keywords:** HIV, treatment adherence and compliance, youth, social support, mHealth, medication adherence, mobile phone

## Abstract

**Background:**

Youth living with HIV (YLHIV) enrolled in HIV treatment experience higher loss to follow-up, suboptimal treatment adherence, and greater HIV-related mortality compared with younger children or adults. Despite poorer health outcomes, few interventions target youth specifically. Expanding access to mobile phone technology, in low- and middle-income countries (LMICs) in particular, has increased interest in using this technology to improve health outcomes. mHealth interventions may present innovative opportunities to improve adherence and retention among YLHIV in LMICs.

**Objective:**

This study aimed to test the effectiveness of a structured support group intervention, Social Media to promote Adherence and Retention in Treatment (SMART) Connections, delivered through a social media platform, on HIV treatment retention among YLHIV aged 15 to 24 years and on secondary outcomes of antiretroviral therapy (ART) adherence, HIV knowledge, and social support.

**Methods:**

We conducted a parallel, unblinded randomized controlled trial. YLHIV enrolled in HIV treatment for less than 12 months were randomized in a 1:1 ratio to receive SMART Connections (intervention) or standard of care alone (control). We collected data at baseline and endline through structured interviews and medical record extraction. We also conducted in-depth interviews with subsets of intervention group participants. The primary outcome was retention in HIV treatment. We conducted a time-to-event analysis examining time retained in treatment from study enrollment to the date the participant was no longer classified as active-on-treatment.

**Results:**

A total of 349 YLHIV enrolled in the study and were randomly allocated to the intervention group (n=177) or control group (n=172). Our primary analysis included data from 324 participants at endline. The probability of being retained in treatment did not differ significantly between the 2 study arms during the study. Retention was high at endline, with 75.7% (112/163) of intervention group participants and 83.4% (126/161) of control group participants active on treatment. HIV-related knowledge was significantly better in the intervention group at endline, but no statistically significant differences were found for ART adherence or social support. Intervention group participants overwhelmingly reported that the intervention was useful, that they enjoyed taking part, and that they would recommend it to other YLHIV.

**Conclusions:**

Our findings of improved HIV knowledge and high acceptability are encouraging, despite a lack of measurable effect on retention. Retention was greater than anticipated in both groups, likely a result of external efforts that began partway through the study. Qualitative data indicate that the SMART Connections intervention may have contributed to retention, adherence, and social support in ways that were not captured quantitatively. Web-based delivery of support group interventions can permit people to access information and other group members privately, when convenient, and without travel. Such digital health interventions may help fill critical gaps in services available for YLHIV.

**Trial Registration:**

ClinicalTrials.gov NCT03516318; https://clinicaltrials.gov/ct2/show/NCT03516318

## Introduction

Globally, young people aged 15 to 24 years account for more than 30% of new HIV infections, over 80% of whom live in sub-Saharan Africa (SSA); AIDS remains the leading cause of death among youth in SSA [1-6]. Despite a lack of age-disaggregated data on antiretroviral therapy (ART) coverage for youth, available data show that youth living with HIV (YLHIV) enrolled in HIV care experience higher loss to follow-up and suboptimal treatment adherence compared with younger children or adults [7-10]. YLHIV face many challenges to effective treatment, including stigma and fear of disclosure to others, lack of social support, lack of services designed to comprehensively meet their unique needs, and limited knowledge about the disease [7-10]. To achieve the Joint United Nations Programme on HIV and AIDS 95-95-95 goals by 2030, understanding and addressing the unique needs of YLHIV will be critical [11].

Although YLHIV experience poorer health outcomes, few interventions to improve these outcomes target youth specifically. Interventions to improve HIV-related outcomes implemented in low- and middle-income countries (LMICs) largely target adults and aim to improve ART adherence; fewer interventions target retention in care [12-14]. Support groups to improve health outcomes among people living with HIV (PLHIV), including YLHIV, are supported by some evidence to improve retention in care [15-22].

Expanding access to mobile phone technology has increased interest in using this technology to improve health outcomes. Digital health interventions such as mobile reminders and interactive voice or SMS responses have shown some effectiveness in improving adherence or retention among PLHIV in LMICs [23-31]. Although research has not examined these interventions specifically among YLHIV in LMICs, preliminary evidence in high-income countries suggests that such interventions are feasible and may impact ART adherence [32-35]. Furthermore, 2 recent studies in South Africa integrated social media into interventions for YLHIV to improve social support and found them to be acceptable and feasible [36,37]. Increasing internet use, feature phone, and smartphone availability in LMICs makes these interventions possible. Developing and testing interventions to improve YLHIV outcomes in SSA is urgently needed, and digital health interventions may present innovative opportunities to improve adherence and retention among youth in LMICs [12-14,38-40].

Nigeria is experiencing a generalized HIV epidemic and has the second largest global burden of the disease, with an estimated 3,438,442 people currently living with HIV [41]. As with other countries in the region, YLHIV in Nigeria experience high loss to follow-up from HIV treatment services, with the greatest losses occurring early in treatment [39,42]. Given increasing access to and use of mobile phone technology in Nigeria, digital health strategies have potential to help meet YLHIV informational and social support needs [43,44].

In response, we developed a structured support group intervention— Social Media to promote Adherence and Retention in Treatment (SMART) Connections—delivered through a social media platform. The intervention’s aim is to improve HIV-related knowledge, social support, and ultimately, retention in HIV treatment and ART adherence among YLHIV. We conducted workshops with local stakeholders and youths to inform intervention design, followed by a feasibility and acceptability study with 41 adolescents aged 15 to 19 years in south-central Nigeria (results published elsewhere) [45]. In this study, we set out to test the effectiveness of the SMART Connections intervention on HIV treatment retention among youth aged 15 to 24 years. We also examined the effects of SMART Connections on secondary outcomes of ART adherence, HIV knowledge, and social support.

## Methods

### Design

We conducted a two-arm, parallel, randomized controlled trial. Participants were individually randomized in a 1:1 ratio to either the SMART Connections intervention (intervention group) or standard of care (control group). This was an open-label study with no blinding of the study staff or participants. We conducted face-to-face structured interviews with participants and extracted data from medical records at enrollment (baseline) and again at the completion of the intervention period (endline), approximately 6-9 months after enrollment. We also conducted in-depth interviews (IDIs) at endline with 2 subsets of intervention group participants: participants with high group participation and those with low group participation (classified by group facilitators). We obtained informed consent from youth aged 18 years and older; for youth aged 15 to 17 years, we obtained parental permission and adolescent assent at enrollment. This study was reviewed and approved by FHI 360’s Protection of Human Subjects Committee, the University of Uyo Teaching Hospital’s Institutional Health Research Ethical Committee, and the Cross River State Health Research Ethics Committee.

### Intervention Description

**Figure 1:**
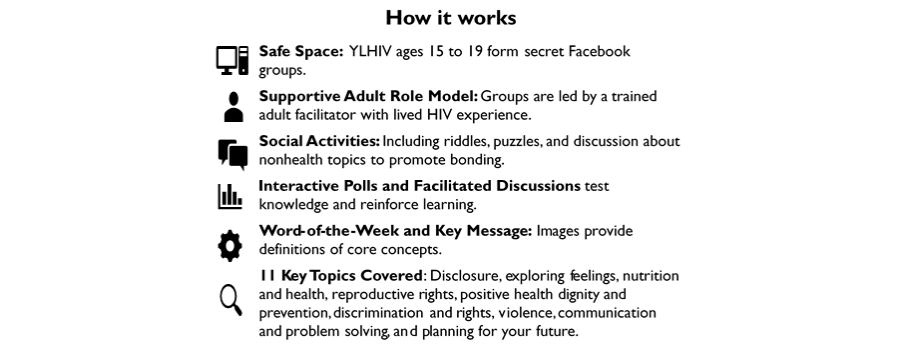
Intervention design overview. YLHIV: youth living with HIV.

SMART Connections was informed through workshops conducted with stakeholders and YLHIV in Akwa Ibom State, Nigeria, to gather input for design and content [12,13]. The intervention was designed to promote retention in HIV care by leveraging social support and improving HIV-related knowledge and treatment literacy [46,47]. The content of the structured support groups was adapted from an existing support group guide, Positive Connections, and delivered through secret Facebook groups [48]. The intervention was delivered over approximately 22 weeks (2 weeks per session) to groups of about 15 to 25 youths, with nearly daily activities (Figure 1). The intervention guide is attached as a Multimedia Appendix 1.

Two community-based organizations were engaged to recruit support group facilitators and assist study staff in training them to deliver the intervention. Selected facilitators had received prior training to facilitate in-person support groups and were living with HIV themselves. Facilitators underwent a 5-day training and received an implementation guide, smartphone and monthly data allowance. Facilitators met monthly with the study staff to debrief on challenges they had encountered and provided support to one another.

Once a sufficient number of participants were recruited and randomized to form a support group, each group began with an in-person meeting, during which participants met one another and the facilitator and agreed-upon ground rules for participation. Facilitators also instructed participants on how to secure their phones (using a password or passcode), turn off Facebook notifications so that messages and posts would not show up on their phones and to log out of Facebook after each use to keep others from accessing their Facebook accounts.

### Standard of Care Services

All study participants continued to receive standard services available to YLHIV in study facilities and communities. Standard health services included routine clinical care for HIV treatment, including viral load tests, active case management by community volunteers with intensive adherence support during the first 4 weeks of ART, and enhanced adherence counseling for patients with unsuppressed viral loads.

All study participants received a smartphone (valued at US $65) and a monthly data bundle (US $3.5 equivalent to 1 gigabyte of data) for the duration of the intervention.

### Study Setting, Population, and Eligibility

The study was implemented in 14 health facilities located in Akwa Ibom State and Cross River State. The 2 states lie adjacent to each other in south-central Nigeria and share many sociocultural similarities. The population of interest was YLHIV aged 15 to 24 years who had been on ART for less than 12 months. In addition to age and ART eligibility criteria, participants had to demonstrate basic literacy for web-based chats, which was assessed at enrollment by asking the participant to read 3 short messages from intervention materials. We excluded individuals who were unable to attend the initial intervention group meeting if randomized to the intervention group, who were enrolled in another research study related to HIV retention or ART adherence, or who were severely ill and unable to provide informed consent at the time of recruitment. For IDIs, facilitators identified group members with high and low participation from whom participants were purposively selected to achieve representativeness by group participation, sex, and age.

### Study Power

The study was powered to detect at least a 0.125 difference in the cumulative probability of retention at endline (0.45 in the control group and 0.575 in the intervention group), corresponding to a hazard ratio of 0.69, with 80% power and 5% significance level for a two-sided comparison using the log-rank test. This resulted in a total sample size of 500 (250 per study arm). Calculations also assumed exponential times to event and a 10% loss to study follow-up. Our estimate of retention was based on program data for the prior year from the United States Agency for International Development (USAID)–funded bilateral HIV services delivery (Strengthening Integrated Delivery of HIV/AIDS Services; SIDHAS) project, which supported HIV services in the facilities selected for this study.

### Changes to the Study Protocol and Implementation

We originally planned to recruit YLHIV aged 15 to 22 years on ART for 6 months or less and expected to achieve our sample size within 3 months. Due to substantially lower than expected numbers of YLHIV enrolling in HIV treatment, we amended the study protocol and expanded the eligibility criteria to include YLHIV aged up to 24 years and on ART for 12 months or less. We also extended enrollment from 3 to 8 months and added 3 facilities (originally 11).

Finally, we intended to collect outcome data from participants after the intervention was completed and at 1 year from enrollment (approximately 6 months later) but were unable to collect a second round of outcome data because of prolonged recruitment—the funding project came to an end before the final round of data collection could take place.

### Recruitment and Randomization

Eligible participants were sequentially recruited from patients attending clinic visits at the study facilities. Health care staff informed eligible participants of the study and directed those interested to a data collector who was stationed in the facility. Data collectors met with potential participants in a private setting in the facility, provided additional details on the study, then proceeded to obtain informed consent and enroll participants. Data collectors assigned participants to study arms using randomization envelopes. Randomization groups were concealed in sequentially numbered sealed opaque envelopes. The allocation sequence was generated using permuted blocks and stratified by local government areas (LGA), which are administrative subunits of the state, by a biostatistician otherwise uninvolved in the study using a validated SAS macro (SAS version 9.4) [49]. Recruitment occurred at health facilities, but randomization was managed from a central location for all facilities within an LGA. This was an open-label study with no blinding of the study staff or participants.

### Measures

We collected demographic information (sex, age, relationship status, education, occupation, and religion) and information on HIV history, including date of HIV diagnosis, ART start date, disclosure of HIV status to others, and viral load testing. Additionally, we collected data on secondary outcomes of ART adherence, HIV-related knowledge, and social support as well as psychosocial factors associated with poor retention, including social isolation, depression, and perceived and experienced stigma.

Retention: For our primary time-to-event analysis, we computed the time retained in care from study enrollment to the date the participant was no longer classified as active on treatment, consistent with the President’s Emergency Plan for AIDS Relief (PEPFAR) indicator definitions [50]. We recorded dates for all scheduled clinic visits from patient medical records for each participant from study enrollment until the end of the study. If a participant failed to return after a scheduled visit for more than 28 days, the date of the missed visit was the date of loss to care recorded, unless death or transfer of service was documented before the missed visit. For a small number of patients, their first missed scheduled visit was scheduled on or within 28 days before study enrollment. If the participant missed this first scheduled visit by more than 28 days, he or she was assigned a retention time of 0.

We also reported on treatment status at endline. To be considered active on treatment at endline, an individual must have attended a visit or had a follow-up visit scheduled within 28 days of the date of their endline questionnaire. For those who did not complete an endline questionnaire, an approximate endline date was used (based on the median time in the study of those enrolled the same month). Participants who died or transferred facilities were categorized accordingly.

Measurement of secondary outcomes and other psychosocial variables are summarized in Table 1.

### Data Collection

Structured questionnaires programmed into password-protected computer tablets were used to collect data from participants at both baseline and endline. We also extracted medical record data (MRE) from patient charts at both time points into a separate form programmed into the tablets. Data from tablets were uploaded daily to a secure computer server. Trained interviewers administered a semistructured IDI guide. IDIs were audio recorded and transcribed verbatim.

### Data Analyses

All analyses used an intent-to-treat approach, in which all randomized participants were included in the statistical analysis and analyzed according to the group to which they were originally assigned, regardless of the treatment they received during the study. The primary hypothesis was that *YLHIV enrolled in HIV treatment services who participate in SMART Connections will be more likely to be retained in HIV care than YLHIV enrolled in HIV treatment services who do not participate in the intervention.*

Kaplan-Meier cumulative retention probabilities are reported with 95% CIs and plotted by study arm. Participants who were confirmed to have died or transferred to a facility outside the study facilities were considered censored. Participants who had elected to drop out of the study (n=8), or for whom all MRE were missing because of missing charts (n=14) or all visit data were missing data in charts (n=3), were excluded from the analysis.

The retention probabilities between the groups were compared using a log-rank test stratified by LGA with a two-sided α of .05. We also report on retention descriptively, examining lapses in care, and return to treatment over the course of study follow-up. The relationships between treatment exposure and secondary outcomes (ART adherence, HIV knowledge, and social support) as well as social isolation, depression, and HIV-related stigma were explored with *t* tests for continuous outcomes and chi-square test for categorical outcomes, using two-sided tests, with a significance level of 0.05.

We applied thematic analysis to the IDI data. A structured codebook was developed *a priori* based on the interview guide; emergent thematic codes were added during the analysis. To assess intercoder consistency, 2 analysts independently coded 3 transcripts, compared coding, and resolved differences through discussion. Coding was conducted using NVivo 12 [56]. Once all transcripts were coded, textual coding reports were produced.

**Table 1:** Secondary outcomes and related psychosocial variables and their measurement.

Concept	Measurement
HIV knowledge and treatment literacy	A set of 14 knowledge-based questions covering HIV transmission, diagnosis, treatment, and treatment monitoring based on topics covered in the SMART^a^ Connections curriculum. Each item is scored 1 if answered correctly and 0 if answered incorrectly. A total knowledge score was calculated based on the proportion of items correctly answered.
Social support	Medical Outcomes Study-Social Support Survey, a 19-item scale that covers the dimensions of emotional, information, affectionate, and tangible social support in addition to positive social interaction [51]. Scores for each item were summed, then averaged. Range of possible scores: 0-5, with a higher score indicating a greater level of social support.
Adherence to antiretroviral treatment	Self-report using the AIDS Clinical Trials Group adherence questionnaire [52]. For these analyses, we limited our analysis to a single item that recorded self-reported ART^b^ adherence for the 3 days before the interview. Values included 0 for no missed doses, and 1 for 1 or more missed doses.
Social isolation	4-item PROMIS^c^ Social Isolation Scale [53]. Participants are asked how often they feel each of 4 different situations occurs in their lives. Responses options include 1=Never, 2=Rarely, 3=Sometimes, 4=Usually, and 5=Always. For this analysis, the total score was a sum of the responses for the 4 items.
Depression	Stanford Patient Education Research Center’s PHQ-8^d^ [54]. The PHQ-8 asks respondents on how many days over the prior 2 weeks they experienced 8 possible symptoms, with response options of *not at all*=0, *a few days*=1, *more than half the days*=2, and *most all of the days*=3. The score for each item is summed, and a total score that ranges from 0 to 24 is assigned. Respondents with scores <10 are classified as not depressed. Respondents who score 10-19 points are considered to have major depression, and those who score 20 or more have severe depression [54]. For these analyses, a categorical variable was created: 1=no depression, 2=major depression, and 3=severe depression.
HIV-related stigma	12-item scale adapted by Reinius and colleagues from the 40-item HIV stigma scale [55]. This shortened stigma scale covers 4 dimensions of stigma: personalized stigma, disclosure concerns, concerns about public attitudes, and negative self-image. Each item is scored on a 4-point Likert-type scale, and the scores added within dimensions with possible scores ranging from 3 to 12 [55]. A higher score indicates a greater level of perceived HIV-related stigma.

^a^SMART: Social Media to promote Adherence and Retention in Treatment.

^b^ART: antiretroviral therapy.

^c^PROMIS: Patient-Reported Outcomes Measurement Information System.

^d^PHQ-8: Patient Health Questionnaire Depression Scale.

## Results

### Overview

**Figure 2:**
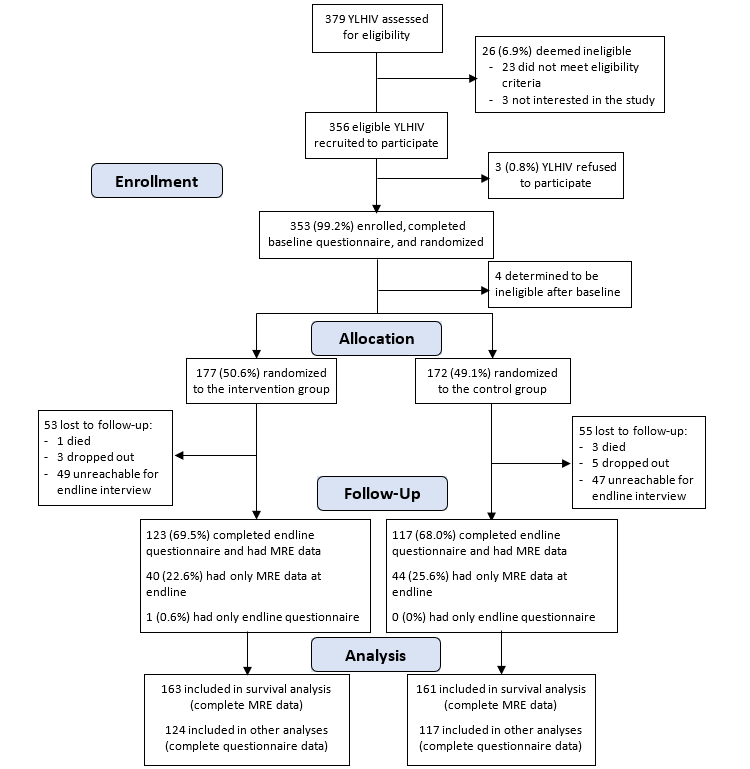
Study flowchart. MRE: medical record data; YLHIV: youth living with HIV.

We recruited 356 youths between September 2018 and April 2019, 353 (99.2%) of whom enrolled in the study (Figure 2). A total of 4 participants were removed after enrollment, as they provided false eligibility information, and were deemed ineligible. Participants were then randomly allocated to the intervention group (n=177) or control group (n=172). At endline (June to November 2019), 108 participants were lost to follow-up from the study, including 4 who died, 8 who discontinued study participation, and 96 who were not reachable for an endline interview. Endline interviews and MRE data were completed for 241 participants. MRE data were collected for an additional 84 participants who were not reachable for an endline interview but who had not elected to END study participation. We conducted IDIs with 21 intervention participants following the endline survey, of whom 16 were female and ranged in age from 17 to 24 years; 13 were classified as high participation and 8 as low participation.

A total of 10 randomization errors occurred during the study: 6 participants randomized to the control group were erroneously recorded by data collectors to be the intervention group and took part in the intervention; 4 participants randomized to the intervention group were erroneously recorded as in the control group and did not participate in the intervention. Two other participants, randomized to and recorded as in the control group, were erroneously contacted to participate in the intervention. In addition, 14 people randomized to the intervention group chose not to participate in the intervention. Thus, a total of 167 participants joined an online support group during the study: 160 randomized to the intervention group, 5 due to randomization errors, and 2 control group participants erroneously contacted.

To assess the potential effects of loss to follow-up, we examined differences between those who did and did not complete an endline interview. There were no statistically significant differences in any background characteristic or baseline values of secondary outcomes between those who completed endline interviews and those lost to study follow-up (results not shown).

### Background

Both study arms were similar in all baseline demographic characteristics (Table 2). Most participants were female 87.7% (306/349) and had completed some secondary school or more; the mean age was 21 years (SD 2.3). More than half of the respondents reported being married or in a relationship. Among those in a relationship (including married), fewer than half reported that their partner knew their HIV status, and about half knew their partner’s HIV status. Most participants reported having access to a mobile phone in their household (303/349, 86.8%), with 67.1% (234/349) reporting that they owned their own mobile phone.

**Table 2:** Background characteristics of study participants at baseline (N=349).

Characteristic		Intervention (n=177)	Control (n=172)
**Sex, n (%)**			
	Female	151 (85.3)	155 (90.1)
	Male	26 (14.7)	17 (9.9)
Age (years), mean (SD)		21.3 (2.3)	21.0 (2.3)
**Relationship status, n (%)^a^**			
	Married	37 (20.9)	40 (23.3)
	Unmarried or in a relationship	83 (46.9)	85 (50.3)
	Single	57 (32.2)	44 (26.0)
**Among those married or in a relationship, n (%)**			
	Partner knows participant’s HIV status^b^	42 (35.3)^c^	57 (45.6)^d^
	Partner has HIV^e^	20 (16.7)^c^	26 (30.0)^d^
	Partner does not have HIV	39 (32.5)^c^	40 (32.3)^d^
	Does not know partner’s HIV status	61 (50.8)^c^	58 (46.8)^d^
**Education level, n (%)^e^**			
	Primary or less	13 (7.3)	30 (17.5)
	Secondary	136 (76.8)	121 (70.8)
	Any postsecondary	28 (15.8)	20 (11.7)
Currently working, n (%)		73 (41.2)	69 (40.1)
**Religion, n (%)**			
	Protestant	152 (85.9)	150 (87.2)
	Catholic	22 (12.4)	15 (8.7)
	Other (all Christian denominations)	3 (1.7)	7 (4.1)
Time on ART^f^ at enrollment (months), mean^g^		4.5	4.5
**Pre-ART WHO^h^ stage, n (%)^i^**			
	Stage 1	93 (60.8)	85 (57.4)
	Stage 2	35 (22.9)	42 (28.4)
	Stage 3	23 (15.0)	20 (13.5)
	Stage 4	2 (1.3)	1 (0.7)
Had access to a phone in the home, n (%)		156 (88.1)	147 (85.5)
Owns a mobile phone, n (%)		118 (66.7)	116 (67.4)
**Ever used social media sites of those who ever use the internet, n (%)**			
	Facebook	101 (57.0)	76 (44.2)
	WhatsApp	69 (39.0)	52 (30.2)
	Instagram	30 (16.9)	14 (8.1)
	Snapchat	13 (7.3)	13 (7.6)
	Other (Twitter, Tinder, Imo, etc)^b^	7 (4.0)	5 (2.9)

^a^3 missing from the control group.

^b^1 missing from the intervention group.

^c^n=120*.*

^d^n=125.

^e^1 missing from the control group.

^f^ART: antiretroviral therapy.

^g^15 missing from the intervention group, and 17 missing from the control group.

^h^WHO: World Health Organization.

^i^24 missing in the control group, and 24 missing in the intervention group.

### Retention in HIV Care

**Figure 3:**
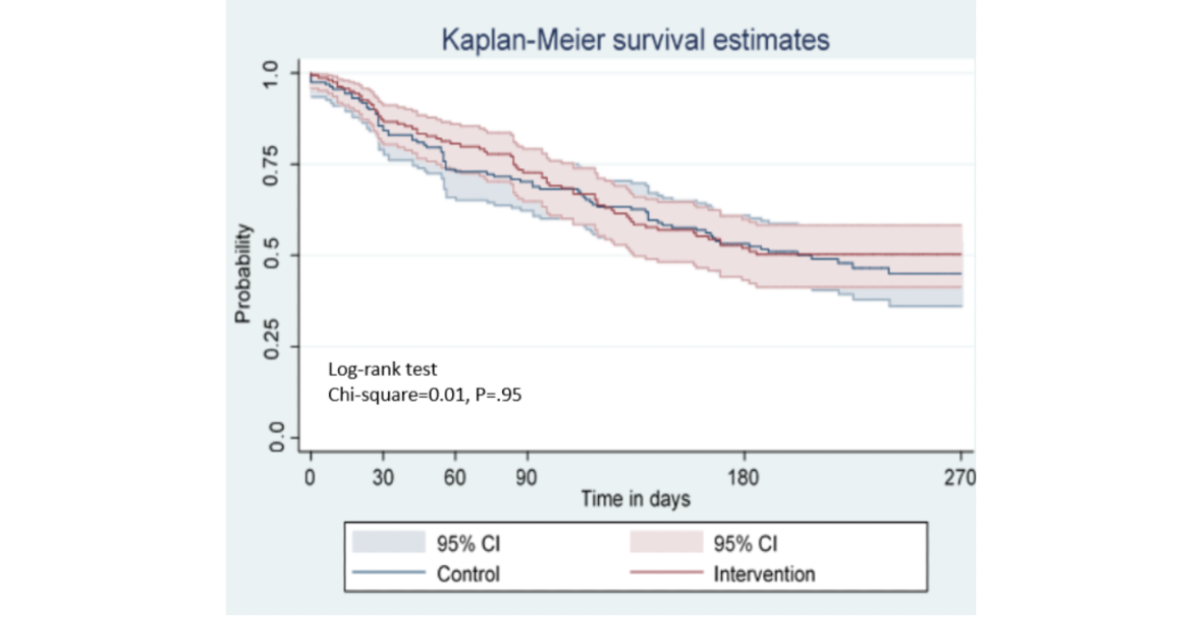
Cumulative probability of retention in care (n=324).

The probability of being retained in HIV care, defined as not having missed a scheduled appointment by more than 28 days, was similar between the intervention and control groups (Figure 3).

The probability of remaining in care without more than a 28-day gap was slightly higher in the intervention group than in the control group at each time point, except at 120 days; however, 95% CIs overlapped between the 2 study arms at all time points (Table 3).

**Table 3:** Probabilities of remaining in care, without a gap of more than 28 days at 30, 60, 90, 180, and 270 days (n=324; 163 intervention and 161 control).

Time		Total at risk	Failures	Probability of being retained in care	SE	95% CI	
						Lower limit	Upper limit
**Intervention**							
	0	163	1	0.99	0.01	0.96	1.00
	30	129	21	0.87	0.03	0.80	0.91
	60	116	9	0.81	0.03	0.73	0.86
	90	102	11	0.73	0.04	0.65	0.79
	180	59	27	0.52	0.04	0.43	0.60
	270	8	2	0.50	0.04	0.41	0.58
**Control**							
	0	161	4	0.98	0.01	0.94	0.99
	30	132	25	0.84	0.03	0.78	0.89
	60	108	17	0.73	0.04	0.65	0.79
	90	103	4	0.70	0.04	0.62	0.77
	180	72	24	0.53	0.04	0.45	0.61
	270	12	8	0.45	0.04	0.36	0.54

Retention, measured as not having missed any scheduled visit by more than 28 days, did not reflect the participant’s treatment status at endline (Table 4). Almost half of all participants missed at least one scheduled visit by more than 28 days during the 6 to 9 months of follow-up and were classified as lost to care at some point; however, most participants were active on treatment at endline. A total of 40.0% (65/163) in the treatment arm and 46.0% (74/161) in the control arm were counted as having been lost-to-care in the time-to-event analysis but were active on treatment at the end of the study.

**Table 4:** Clinic visits and retention over the study period for participants included in the retention analysis, intention to treat (n=324: 163 intervention and 161 control).

Characteristics		Intervention (n=163)	Control (n=161)
Number of clinic visits during study, mean (range, SD)		3.4 (0-10, 1.9)	3.9 (0-10, 1.9)
Missed at least one visit by >28 days, n (%)		70 (42.9)	78 (48.5)
**Time between scheduled visits (months), n (%)^a^**			
	1	111 (72.1)	118 (75.6)
	2	33 (21.4)	30 (19.2)
	3	7 (4.5)	5 (3.2)
	4	1 (0.6)	2 (1.3)
	>5	2 (1.3)	1 (0.6)
**Treatment status at endline, n (%)^b^**			
	Active on treatment	112 (75.7)	126 (83.4)
	Lost to care	22 (14.9)	15 (9.9)
	Transferred out	13 (8.8)	7 (4.6)
	Deceased	1 (0.7)	3 (2.0)
Of those active on treatment at endline, n (%) who missed at least one scheduled visit by >28 days		45 (40.2)	58 (46.0)

^a^9 missing from intervention group, and 5 missing from control group.

^b^15 missing from intervention group, and 10 missing from control group.

The average number of clinic visits during the study varied by participant, in part because of variation in the length of time between scheduled visits. The average time between schedule visits was 1 month for most, but one-quarter of participants had an average time between visits ranging from 2 to more than five months.

Barriers to clinic attendance, as described in IDIs, included traveling away from home, anticipating stigma at the clinic, and competing obligations. When asked how easy it was to attend scheduled appointments, one participant described it as follows:

When you go to the hospital and they saw you…they will tell people about you, this is the kind of sickness that this person has, so that makes me very difficult to go.

24-year-old female, high participation

A few participants described hesitation leaving school, work, or family to attend appointments if it required disclosing their HIV status. Findings were similar in endline questionnaire open-ended data; among 79 participants who reported missing an appointment by one week or more, reasons for doing so included traveling (33/79, 42%), transportation issues (20/79, 25%), or were busy (13/79, 16.5%).

Most IDI participants described that the intervention impacted their attitudes and behaviors toward attending scheduled appointments. Some described that the intervention reinforced the importance of attending appointments to prevent gaps in medication and taught them to “take your drugs so that you'll feel okay ” (21-year-old female, high participation). Others described learning strategies to avoid missing appointments, such as asking someone to collect refills if they could not. A few reported that the intervention served as a reminder to attend scheduled appointments; one participant mentioned receiving reminders from group members on their appointment day. Another described that being part of the group:

Something was there telling me to always don't miss appointment, to take my drugs, it was important, so I think the group was a reminder.

19-year-old female, low participation

In contrast, a few participants, most categorized as low group participation, felt that the intervention did not affect their retention in care.

### Secondary Outcomes

HIV-related knowledge was high in both groups at endline but significantly higher in the intervention group compared with the control group (Table 5). No other statistically significant differences between study arms were observed for ART adherence, social support, social isolation, HIV-related stigma, or depression.

**Table 5:** Bivariate relationships between treatment group and endline HIV knowledge, psychosocial variables (*t* test), and self-reported adherence (chi-square test) among participants who responded to the endline questionnaire (n=241).

Outcomes		*t* value (*df*)	Chi-sqaure (*df*=1)	*P* value
HIV knowledge score		-2.96 (239)	N/A^a^	.003
Social isolation score		-0.79 (239)	N/A	.43
**Social support score**				
	Total score^b^	-0.95 (238)	N/A	.34
	Tangible subscore	-0.38 (239)	N/A	.70
	Emotional/informational subscore^b^	-0.67 (238)	N/A	.51
	Affectionate subscore^b^	-0.64 (238)	N/A	.53
	Positive social interaction subscore	-1.57 (239)	N/A	.12
**Stigma score**				
	Total score^c^	0.34 (196)	N/A	.73
	Personalized stigma subscore^d^	-0.51 (209)	N/A	.61
	Disclosure concerns subscore^e^	0.77 (235)	N/A	.44
	Concerns about public attitudes subscore^f^	-0.54 (213)	N/A	.59
	Negative self-image subscore^g^	0.02 (229)	N/A	.98
Depression^h^		N/A	0.15	.70
Adherence^b^		N/A	0.32	.57

^a^N/A: not applicable.

^b^1 missing from the intervention group.

^c^23 missing from the intervention group, 20 missing from the control group.

^d^17 missing from the intervention group, 13 missing from the control group.

^e^4 missing from the control group.

^f^13 missing from the intervention group, 13 missing from the control group.

^g^4 missing from the intervention group, 6 missing from the control group.

^h^1 missing from the intervention group, 1 missing from the control group.

Although the 2 study arms did not differ significantly on social support, nearly all IDI participants, including those with low intervention participation, stated they received social support from facilitators and other group members. These participants described receiving encouragement and advice, having people to “share my feelings with” or “someone to talk to,” and receiving answers to factual and personal questions. One participant described the following:

I felt like I was not alone in the journey and it was really cool...it was amazing. I don't know how to say it in words but it's something to build (us up) because sometimes we can't just do it by ourselves. We need to find people in the situations with us for us get stronger, so the group actually made me stronger.

19-year-old female, low participation

Some support was related to self-management, such as encouragement to adhere to ART. For example, one participant described group members as:

…people that would encourage you no matter anything, they tell you no matter anything, that they’re okay with the drugs,… [they] encourage you to take the ART.

22-year-old male, low participation

Participants frequently received multiple types of support and often supported other group members by sharing their own experiences or providing emotional support and advice.

Nearly all IDI participants stated they felt a sense of connectedness with the group, sometimes described feeling as if they were “a family” or that participants got along “as brother and sister.” This feeling was often attributed to having group members of the same age range and HIV status and conferred a sense of safety and confidentiality within the group. In endline questionnaires, when asked what they liked most about the intervention, the most common responses included receiving encouragement and support, the ability to share their problems, and feeling a sense of unity or belonging.

When asked about sources of social support outside of the intervention group, most said they did not receive social support outside of the group, sometimes elaborating that they preferred not to disclose their HIV status to family and friends due to fear of stigma:

I don't like disclosing… I don’t know if that person is a victim of HIV…the person may start broadcasting me. Things like that so that’s why I don’t tell her, I didn’t tell people… They will not be able to encourage me since they don’t know what I’m passing through … I keep it [my status] to myself.

19-year-old male, high participation

Only a couple of IDI participants stated that they received support from family members; a few mentioned a health care provider.

### Participant Perspectives on the Intervention

Nearly all who took part in the intervention (as treated) and completed an endline interview agreed that the intervention was useful to them, they enjoyed the intervention and felt comfortable interacting with the facilitators and other group members, and they would recommend the intervention to other YLHIV (Table 6). Nearly all also reported that connecting to the groups on Facebook was somewhat or very easy.

**Table 6:** Intervention participant perspectives on the web-based intervention at endline (n=127).

Characteristic		Intervention (n=127)^a^
**Agree with the following statements, n (%)**		
	I enjoyed being a member of the online support group^b^	122 (97.6)
	I received information during the support group that was useful to me^b^	124 (99.2)
	Participating in the support group helped me better understand HIV infection^b^	124 (99.2)
	I felt comfortable interacting with other group members^b^	116 (92.8)
	I felt comfortable interacting with the group facilitator^c^	118 (95.2)
	I made new friends in the group^b^	94 (75.2)
	I would like to continue to be part of this group^b^	121 (96.8)
	I think Facebook groups are a good way for young people on ART to interact with each other^b^	124 (99.2)
	I think Facebook groups are a good way for support group leaders to get information to people on ART^d^	119 (99.2)
	I would recommend this Facebook group to other young people living with HIV^b^	121 (96.8)
**Ease of connecting to Facebook group, n (%)**		
	Very easy	80 (63.0)
	Somewhat easy	32 (25.2)
	Somewhat difficult	13 (10.2)
	Very difficult	2 (1.6)

^a^This number includes the 6 participants assigned to the control group who took part in the intervention (as treated).

^b^2 missing.

^c^3 missing.

^d^7 missing.

IDI participants often conveyed appreciation for learning about practical aspects of managing HIV, such as taking ART at consistent times during the day and eating “a balanced diet” to support overall health. Many participants also enjoyed learning why medication adherence is important. Participants recalled learning that ART adherence would help them feel healthier and achieve a longer life. One participant described as follows:

by taking the drugs and eating your food every day, your body will be okay … but if you avoid taking that drugs and you are not doing anything, you don’t go for test, you might die at any point in time and nobody will know the purpose of your death.

22-year-old female, high participation

Many IDI participants also expressed enthusiasm for the social and interactive elements of the intervention, such as riddles posed by the facilitator. Most felt the Facebook platform was acceptable, reporting that the “secret” groups ensured their privacy, and the web-based format allowed them to interact with the group at their convenience. One participant described as follows:

you’ll use it [the group] at the comfort of your home, not pressing you to go out, do this, every time to hear about the new information that you need to learn. Peacefully, you’ll just learn inside your room, inside your, the comfort of your own home. So, I was very happy about that one.

17-year-old male, high participation

## Discussion

The SMART Connections intervention was designed to improve treatment retention among YLHIV by improving HIV-related knowledge and social support. Our findings indicate that the intervention did not significantly improve retention or social support; however, HIV-related knowledge did improve significantly. Data from IDIs provided evidence of perceived improvements in social support. Intervention participants also overwhelmingly found the intervention acceptable, liked the web-based platform, and reported it had helped them in their HIV treatment.

Our study adds to a small yet growing number of studies targeting YLHIV in LMICs. A 2019 literature review identified only 10 studies between 2016 and 2018 focused on adolescents (10-19 years) and/or YLHIV (15-24 years) in LMICs [36]. Among the 10 studies, 5 tested variations of youth-friendly services, 3 examined different community-based services to ALHIV/YLHIV, and 1 examined an SMS reminder system. Similar to our results, half of the studies reviewed—3 studies on youth-friendly services, 1 on community-based adherence clubs, and 1 on SMS reminders—showed no effects on retention [14]. Five studies found significant associations between youth-focused interventions and retention in HIV services; however, all were retrospective cohort studies, with limitations inherent to observational studies [14].

To date, most research on digital health interventions among PLHIV remains focused on high-income country settings, with a few exceptions [57-61]. A 2016 South African study examined the feasibility and acceptability of a web-based social media platform, MXit, to support YLHIV aged 12 to 25 years. Similar to our research, investigators found that the majority of youths in the study (84%) felt that offering a service outside of in-person meetings was useful [37]. A second South African study recently examined using mobile phones to provide peer mentorship for youths newly diagnosed with HIV [62]. This small case-control study also found no differences in 6- and 12-month retention or viral suppression between the groups [62]. Looking forward, interest in the use of digital health interventions to help meet the needs of YLHIV is growing. We identified published protocols of current studies examining interventions targeting youths; however, all 3 are being conducted in the United States [63-65].

SMART Connections was originally designed for adolescents aged 15 to 19 years living with HIV. The practical reality of ART initiation in our study settings, despite being among the highest HIV prevalence in Nigeria, led us to expand the age range for this study [66]. Most study participants were aged >19 years and although much of the content is relevant to the people of all ages, we believe future research should explore if some of the intervention content should be tailored to the differing developmental needs of those aged 15 to 19 years and those aged 20 to 24 years. We also noted that most (88%) of study participants were female. Although HIV prevalence among females in this age group is, on average, twice that of males in the age group, other research has demonstrated that the high ratio of females to males is not unusual in this setting [2]. A 2014 study examining the characteristics of YLHIV initiating ART in Nigeria found that from 2004 to 2013, 92% of new ART patients aged 15 to 24 years were female [42]. Given the high ratio of females to males, it may be worth exploring if and how the intervention could be better tailored to meet the gender-specific needs of young women.

Retention can be difficult to define and measure. Medical records, the source for our primary outcome, posed a particular problem as records contained errors, conflicting information, and missing data. In addition, most participants in care at endline had missed at least one appointment by more than 28 days, suggesting that, although missed visits and ART refills are problematic for effective treatment, they are a reality for many YLHIV, and missing visits does not necessarily translate to categorical loss to follow-up.

Measuring ART adherence is similarly difficult; self-reported gaps in adherence are underreported [67]. We originally considered using viral load as a marker for adherence; however, viral load test results were not widely available for participants, either because testing was not happening or results were not being recorded reliably. The study lacked sufficient resources to pay for testing for all participants. Future research should include viral load as a primary outcome for interventions designed to improve health outcomes among PLHIV.

Although we did not find a statistically significant intervention effect on social support, results from qualitative data indicate that participants developed friendships with, received support from, and grew close to other group participants despite interacting almost entirely on the web. One possible reason for the lack of an intervention effect on social support may have been that the measure we used was not HIV-specific; we may not have captured the type of social support participants felt they received through the intervention. Given challenges related to stigma and disclosure, further research is needed to operationalize and measure social support associated with HIV.

### Limitations

Despite many strengths, including the use of rigorous experimental study design, the study had a number of limitations. Several factors limited the study implementation and perhaps the interpretation of results. First, the results may not be generalizable to other youths outside our study areas. In addition, recruitment lagged, taking more than twice as long as planned because YLHIV enrollment in ART was substantially lower than estimated using HIV service data before the study. Despite protocol modifications, we were only able to enroll 69.8% (349/500) of the planned participants, reducing the study’s power. Slow enrollment also prolonged the time necessary to enroll enough intervention participants to form a support group in many cases. Thus, participants contributed different amounts of time to the study, and some intervention group participants waited months before their support group could begin. This may have added to their risk of loss to follow up before initiating the intervention.

The context of HIV service delivery also changed during the study implementation. A dedicated *surge* in PEPFAR-supported HIV services aimed to increase the number of PLHIV enrolled on treatment and retained in care began in the study sites partway through the study [68]. This surge entailed intensive efforts to better support HIV treatment services and PLHIV in their care and treatment. Strategies including community-based ART initiation, multimonth ART dispensing for stable patients, and community-delivered ART refills were implemented to increase ART initiation and to improve treatment adherence and retention [68]. These changes meant that the average time between clinic visits and the number of visits participants had during the study varied. Although participants in both study arms were exposed to this changing context, retention increased dramatically during the surge; retention at endline was considerably higher in both study arms compared with retention rates indicated by the presurge programmatic data used to calculate effect size estimates. These external efforts may have masked any possible impact that the intervention had on retention.

### Conclusions

Our findings of improved HIV knowledge and high acceptability are encouraging despite a lack of measurable effect on retention. No single psychosocial intervention is likely to meet the varying needs of YLHIV, but the SMART Connections intervention appears to contribute to some of those needs. Digital health support groups may fill critical gaps in the services available for YLHIV. Given the increasing use of social media by youth, the platform may be a preferred resource for some aspects of HIV-related support. Web-based delivery of support group interventions through platforms such as Facebook can permit people to access information and other group members privately, when convenient, and without travel.

Moving forward, we suggest a few adaptations to the SMART Connections intervention and to continue to examine its potential effects. Expanding groups to include YLHIV on ART for more than 1 year may provide better support to those newly initiating treatment and help meet their own informational and support needs. Findings from the IDIs suggest that the intervention had a perceived effect on social support. We believe that the tool we used to measure social support may not have adequately distinguished between more general social support and social support related to HIV. Further work to develop and test measures of social support that better reflect the support given/provided in the context of HIV should be pursued. Finally, focusing on more reliable outcome measures, such as viral load, is strongly recommended for interventions attempting to improve HIV-related health outcomes.

## Appendix

Multimedia Appendix 1 Intervention implementation guide.

Multimedia Appendix 2 CONSORT-eHEALTH checklist (V 1.6.1).

## Abbreviations

ART: antiretroviral therapy
IDI: in-depth interview
LGA: local government area
LMIC: low- and middle-income country
MRE: medical record data
PEPFAR: President’s Emergency Plan for AIDS Relief
PLHIV: people living with HIV
SIDHAS: Strengthening Integrated Delivery of HIV/AIDS Services
SMART: Social Media to promote Adherence and Retention in Treatment
SSA: sub-Saharan Africa
USAID: United States Agency for International Development
YLHIV: youth living with HIV
