# Supplementary material for: A Social Media–Based Support Group for Youth Living With HIV in Nigeria (SMART Connections): Randomized Controlled Trial
Source: J Med Internet Res. 2020 Jun 2;22(6):e18343. doi: 10.2196/18343 (PMC7298637; doi:10.2196/18343)
Supplement: Multimedia Appendix 1 [file jmir_v22i6e18343_app1.pdf]

USAID/YouthPower Action

Social Media to Improve Art Retention and Treatment Outcomes Among Youth Living with HIV in Nigeria (SMART) Connections Program Guide

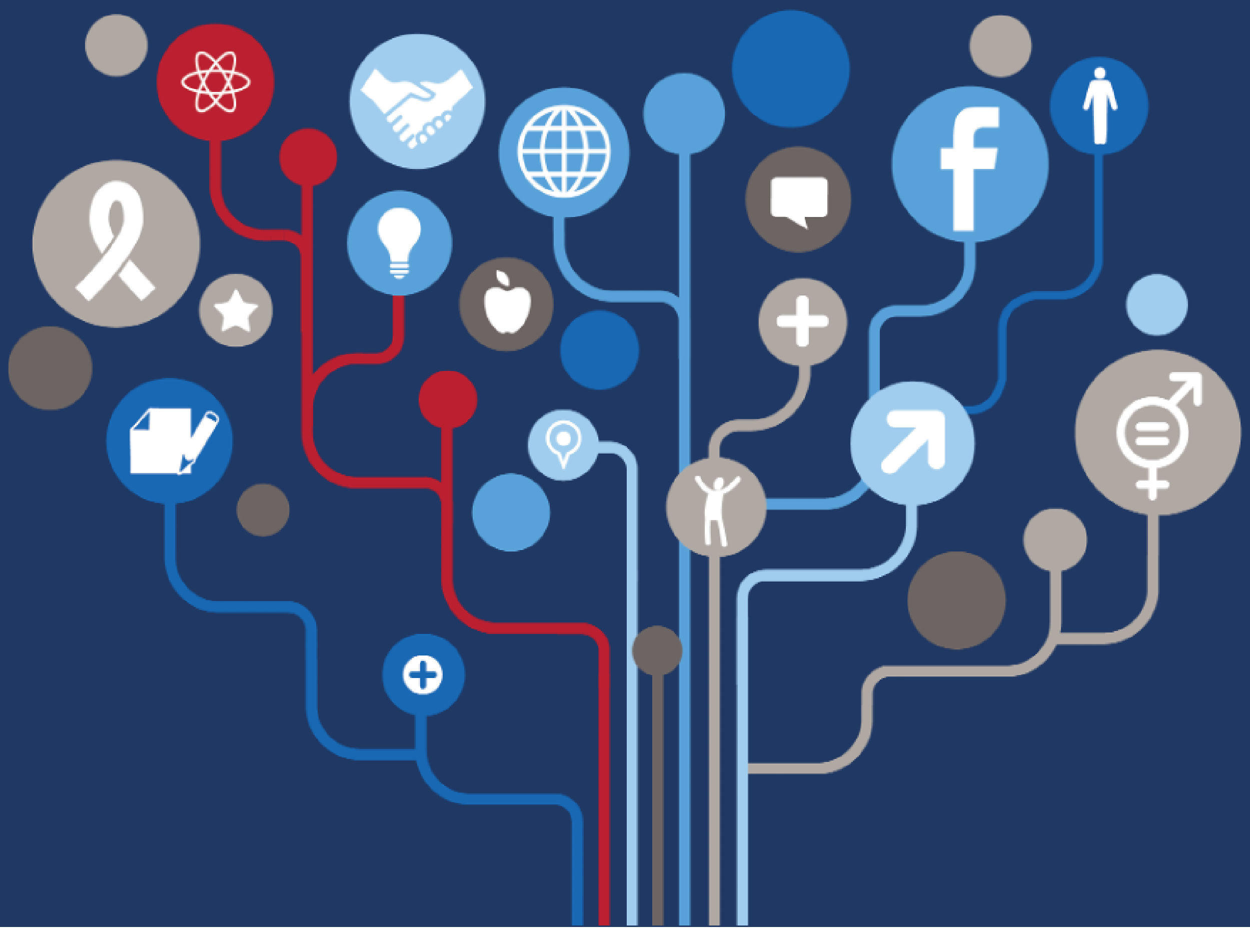

Disclaimer

The viewpoints expressed in this toolkit are those of the authors and do not necessarily represent the official position of the U.S. Agency for International Development, the U.S. President's Emergency Plan for AIDS Relief, or the United States Government.

SMART CONNECTIONS
PROGRAM GUIDE

January 2020

YouthPower Action supports and advances USAID's Youth in Development Policy through evidence-based positive youth development programming across all sectors and country contexts by providing technical assistance to USAID Missions and operating units. YouthPower Action's activities increase youth engagement and youth voice to strengthen USAID's positive youth development programming.

This publication was made possible by the support of the American People through the support of the U.S. President's Emergency Plan for AIDS Relief (PEPFAR) through the U.S. Agency for International Development (USAID) under task order contract number AID- OAA-TO-15-00003, YouthPower Action under IDIQ contract number AID-OAA-I-15-00009, YouthPower: Implementation.

It was prepared by Kate Plourde, Lisa Dulli, Hannah Silverstein, Kate R. Murray, Leila Dal Santo, Kathleen Ridgeway, Catherine Packer, and Donna McCarraher; original artwork by Hannah Silverstein; layout and design by Jill Vitick and Irinn Vinaiphat.

Recommended format for citation

Plourde K, Dulli L, Silverstein H, Murray KR, Dal Santo L, Ridgeway K, Packer C, and McCarraher D. SMART Connections Program Guide. FHI 360; January 2020.

Washington, DC: USAID's YouthPower Implementation, YouthPower Action.

Acknowledgements

Several professionals contributed their time and expertise to the development of this report. We thank the following people for providing technical review and/or input: Kristin Brady, FHI 360; Kristina Morzan Kaplia, FHI 360; Lana Lee, Adult Clinical Branch Bureau for Global Health, Office of HIV/AIDS, Division of Prevention, Care, and Treatment U.S. Agency for International Development (USAID); and Elizabeth Berard, Office of HIV/AIDS, Bureau for Global Health, USAID.

Portions of this toolkit have been adapted and reprinted from previous publications including FHI 360. Positive Connections: Leading Information and Support Groups for Adolescents Living with HIV. 2013. FHI 360: Durham, NC.

CONTENTS

Introduction and Overview iii

Starting a Virtual Support Group v

Pre-Program: In-Person Meeting viii

Session 1: Understanding HIV 2

Session 2: Treatment and Adherence 9

Session 3: Disclosure 16

Session 4: Exploring Your Feelings 24

Session 5: Nutrition and Health 33

Session 6: ReproduCtive Health 41

Session 7: Positive Health, Dignity, and Prevention 49

Session 8: Stigma, DIscrimination, and Rights 57

Session 9: Violence 65

Session 10: Communication and Problem Solving 73

Session 11: Planning for Your Future 80

Citations 88

Annex: Referral Charts 89

INTRODUCTION AND OVERVIEW

Young people living with HIV (YLHIV) face many challenges with adherence to antiretroviral therapy and retention in HIV health care services resulting in high rates of mortality among this population. These challenges include fear of stigma or disclosure to others, lack of social support, and limited knowledge about the disease itself, among many others (1-3). Efforts to improve care and treatment outcomes for YLHIV must begin by providing young people with factual information and social support. Given increasing access to and use of mobile phone technology, digital strategies have potential to support YLHIV and help meet informational and social support needs that could contribute to improved health outcomes (1,2).

SMART (Social Media to promote Adherence and Retention in Treatment) Connections, is an on-line, structured, support group program delivered through "secret" Facebook™ groups by trained facilitators. The program is designed to improve retention in HIV health services and to support antiretroviral treatment (ART) adherence among YLHIV. Informational content provided through this program was adapted from youth development curriculum including FHI 360's Positive Connections guide for Leading Information and Support Groups for Adolescent Living with HIV(4), among others. The program provides participants with opportunities to discuss psychosocial and health-related issues with the facilitator and other group members and integrates key features of positive youth development programs. The program is intended to take place over 22 weeks and covers 11 key topics:

- Understanding HIV
- Treatment and Adherence
- Disclosure
- Exploring your Feelings
- Nutrition and Health
- Reproductive Rights
- Positive Health Dignity and Prevention
- Discrimination and Rights
- Violence
- Communication and Problem Solving
- Planning for Your Future

Within each topic, content formats include:

- At-a-glance images providing and overview of content in each session
- Word-of-the-Week and Key Message images providing definitions of core concepts
- Role Model story cartoons, posted as photo albums and demonstrating positive health behavior
- Interactive polls to test knowledge
- Discussion questions
- Social activities including riddles, puzzles, and discussion about non-health topics to promote bonding

All program image files are available for download from YouthPower.org.

This program was initially developed for and implemented among adolescents and youth living with HIV in Nigeria(3). The materials presented in this guide are intended

to be adapted as appropriate to the local environment, cultural context, and specific needs of YLHIV. Facebook™ was selected as the delivery channel for this program based on input from key stakeholders, including young people themselves, and because of the platform's secret group function.

Who is this guide for?

This guide is intended for use by adults trained in, or with experience in, HIV counseling and testing for YLHIV; leading psychosocial support groups for PLHIV; and/or who provide other psychosocial support services to YLHIV. Group facilitators should have adequate knowledge of HIV infection and treatment. This guide assumes that group leaders will use their own phone to facilitate the program and that group leaders have a working knowledge of how to use their individual phones. Group leaders can, alternatively, use a desktop or laptop computer if available.
Facilitators may include the following groups of people:

- Health care providers, such as nurses, doctors, and community health workers
- Counseling professionals, including psychiatrists, psychologists, therapists, and social workers
- Teachers
- PLHIV
- Parents and caregivers
- Faith-based leaders

STARTING A VIRTUAL SUPPORT GROUP

Your virtual support group might be a stand-alone support group, a supplement to an in-person support group for YLHIV, or it could be part of a broader program of youth activities. Facebook™ is just one of the many social media platforms that could be leveraged to meet the needs of YLHIV. Program managers should identify the social media platform that is most preferred by participants, as well as that provides the greatest level of privacy and security, and adapt this intervention as appropriate.

Who should participate in the information and support groups?

The guide was developed to support the implementation of virtual support groups for young people living with HIV between the ages of 15-24 and enrolled in ART services. YLHIV may include:

- Those who were infected perinatally or as children who have known about their status for a while and who are making the transition from pediatric care and treatment to adolescent and adult services.
- Those who were infected perinatally or as children but who only learned about their status when they became adolescents.
- Those who were infected as adolescents or youth and who are initiating HIV care and treatment.

Virtual support groups may help YLHIV to overcome some of the factors that can make it difficult to attend in-person support group meetings, such as transportation costs, living away from home because of school or work, fear of stigma, or perceived danger associated with disclosure to household members. Because this program is delivered via social media and includes a mix of text messages, photos, and discussion, basic literacy is necessary to participate fully.

Considerations for forming support groups

Program managers will need to make decisions about the appropriate composition and segmentation of support groups for their programs. When determining how groups should be delimited, program managers should consider:

Age of participants: This guide was developed for implementation with YLHIV ages 15-24. Adaptation for, and testing with, very young adolescents (ages 10-14) is recommended prior to implementation with this age group. When implementing with YLHIV between the ages of15-24, program managers should consider how to best divide participants into groups by age band. When developing this intervention, YouthPower Action conducted a feasibility and acceptability assessment with YLHIV between the ages of 15-19; later the project conducted a pilot assessment of the intervention with YLHIV up to age 24. Anecdotal findings point to benefits for each approach. For example, when younger youth are in groups with members closer to their age, they may feel more comfortable participating in discussions. However,

including older youth in the same group as younger youth may provide an opportunity for older youth to act as positive role models as younger youth may appreciate the additional life experience of older group members. Finally, it is important to research laws regarding minors and confidentiality as consent to participate may be required for minors in some countries.

Treatment stage and transmission pattern: It is important to consider group members' treatment stage and how they were infected with HIV when forming groups. For example, perinatally infected youth may be more familiar with the health care system than youth who are newly diagnosed. Each group is likely to face distinct adherence challenges. Program managers may consider creating groups specifically for individuals who are newly diagnosed or creating mixed groups of youth who are at various points in their treatment. As with age, there are benefits to each approach. In addition, the mode of transmission (i.e. at birth, through consensual sexual activity, or through the experience of violence, as some examples) will impact young people's concerns and the specific issues that they need support to address.

Gender: This intervention was tested with mix gender groups; however, gender disaggregated groups may be more appropriate in certain cultural contexts or with particular sub-populations of youth.

Additional vulnerabilities: Certain individuals might require additional counseling before attending the information and support groups. They may be individuals who abuse drugs or alcohol, young people who have been sexually assaulted or abused, and individuals with severe depression or who are suicidal. Program managers should use their best judgment to determine if someone needs more immediate help or intensive counseling before they join (or in addition to joining) the group.

Setting up a "secret" Facebook™ group

Step-by-step information for setting up and managing Facebook™ groups are available on the Facebook™ website: https://www.facebook.com/help/1629740080681586/?helpref=hc_fnav. To establish ground rules, support participants to maximize privacy settings, and to introduce the program approach we recommend an initial in-person meeting to set up groups. Guidance for this meeting appears later in this document.

Special considerations and additional resources for facilitating a Facebook™ group

Ensuring confidentiality is critical for all people living with HIV but is especially important for young people who may be more vulnerable to the negative consequences of disclosure. While disclosing one's status to people who are trustworthy and supportive has many benefits, there may also be risks. These risks include stigmatization and discrimination, emotional and/or physical violence, and, in some cases, death. It is always the person living with HIV's decision to whom they choose to disclose and when to do so.

Participation in any support service for people living with HIV carries a risk of disclosure—for example, a neighbor may see a young person walking into an HIV clinic and assume that he/she is HIV positive and tell others. The risks of participation in a Facebook™ group for people living with HIV are like those for someone participating

in any other support services; however, there are some unique considerations. To ensure that participation in the Facebook™ group is kept confidential, this program was designed for implementation via "secret groups."

All participants should be provided information about online safety and how to keep their online activities private. Furthermore, all group members must commit to keeping their own and their peers' information confidential.

Remember that YLHIV are particularly vulnerable to violence and poor mental health outcomes. In addition to typical youth development programs, ensure that your referral network includes child protection services, gender-based violence response programs, and national suicide prevention initiatives, as they exist in your country or community. It is important that support group facilitators, are aware of the additional resources available in their area—facilitators can use the "Developing a Referral Network" template located in Annex 1 to outline available resources.

In addition to facing stigma and discrimination in their homes, schools, or communities; young people using social media face the risk of cyber bullying. Cyber bullying is bullying that takes place online, and it can have serious consequences. If participants experience any type of bullying because of participation in this program or otherwise, you must be prepared to respond. Facebook™ developed a set of resources for identifying and responding to cyber bullying. These resources are available via the Facebook™ Bullying Prevention Hub: https://www.facebook.com/safety/bullying.

Finally, remember that support group facilitators will play an important role in the lives of YLHIV. Sometimes, however, participants may have needs that are outside of the purview of facilitators' role to address. It is critical that program managers work with facilitators to set boundaries and clearly delineate the responsibilities and expectations of their role. For example, participants may reach out to facilitators for support outside of the group platform; they may request phone calls or send private messages. Depending on how your program is structured, some facilitators may play a more active role in supporting patient retention. Participants may request financial support for their families. It is important that programs consider the potential additional costs of this type of support (for example the cost of facilitator transportation costs or airtime) and set clear protocols describing what the facilitator is responsible for and what financial support the program can provide. This may be different for every program.

PRE-PROGRAM:
IN-PERSON MEETING

Each virtual support group is required to have one initial in-person meeting to introduce the program to participants, establish group agreements, and provide guidance on using Facebook™. Follow the steps below to lead the meeting:

1. **Welcome participants:** Introduce yourself and welcome participants to the in-person meeting and virtual support group. Ask everyone to introduce themselves by sharing their name and one activity they participate in that brings them joy.

2. **Explain the purpose of the group and how it will be used in the following weeks.** Review the program format. Explain to participants that each day the facilitator will post new materials to the group page. Explain that participants will get more out of the program if they engage in conversation by responding to discussion questions and each other's posts. Explain that while there might be some variation in the agenda, in general, every session will include an overview of key messages, a word-of-the week, group discussion questions, social activities, and photo albums sharing stories and key information.

3. **Establish group agreements or ground rules***:* One of the first things you and your group should do is to create and agree upon some ground rules to keep the meetings productive and respectful. Allow the participants to come up with their own list of agreements or rules while you record them on a chalkboard, white board, or flip chart. Make sure the rules include some form of the following:

    - Respect for ourselves
    - Respect for each other (maintaining confidentiality and supporting each other, no stealing, no gossiping, no vulgar language, no taking advantage of younger members, listening to others when they are talking, being considerate of each other's feelings, sharing equally with others)
    - Respect for the group (good participation and contribution each week, listening to facilitators, no fighting or arguing with others in the group)
    - If confidentiality is mentioned and written as one of the ground rules, draw a circle around it. If not, write "Confidentiality" in large letters on the flip chart. Explain that it is important that participants feel comfortable to share their thoughts. Some may decide to share personal experiences. A commitment to confidentiality within the group will help everyone feel more comfortable sharing their thoughts.
    - Take a photo of the list and upload it as a file to the Facebook™ group page. Participants will be able to refer to these ground rules over the course of the program.

    Discuss, and determine together as a group, the consequences that group members will face if ground rules are broken. For example, participants should decide what would happen to a member who allows others to read posts to the

secret group or revels a group members' HIV status to someone outside of the group.

4. **Allow participants to pick a name for the group:** Consider allowing participants to vote for a virtual support group name. This will allow participants to feel some ownership of the group. Use the chalkboard, white board, or flip chart to list all the group name suggestions. Then ask participants to vote for their favorite group name.

5. **Discuss your role as facilitator:** Describe your role as facilitator and group moderator. Explain to participants that they can reach out to you at any point during the week either through Facebook™ (by sending you a private message) or by phone. Provide your contact information as well as your supervisor or program manager's contact information.

6. **Demonstrate use of Facebook™ group on phone:** Invite each participant to the secret Facebook group. Once you have added everyone, ask participants to log onto Facebook using their mobile phones. Walk through the different Facebook group features with participants. Stop frequently to make sure participants are following along and ask participants if they have any questions. Make sure you review the following topics:
   - How to create a Facebook™ account (for those who do not already use Facebook™)
   - How to access the Facebook™ group page
   - How to write a post and how to comment on other participants' posts
   - How to upload files and photos
   - How to create and participate in polls
   - How to use Facebook™ Messenger (make sure all participants have downloaded Messenger to their phones)

7. **Discuss safety measures:** Explain to the group that while this Facebook group will be closed and "secret," and that is not open to people who are not invited by the facilitator, we will all need to take special precautions to protect both our own information and the information that other group members share.
   - Ask the group to brainstorm strategies to ensure that people don't see what they've been looking at on their phone and are not able to access the group page. Make sure the suggestions include some form of the following:
     - Sign out of Facebook™ after each use
     - Don't give out passwords
     - Use a nonpredictable password
     - Don't store passwords on the phone
   - Explain to the group that it is important to ensure that they are using the Internet and social media safely. Ask the group to brainstorm strategies to stay safe online. Make sure the suggestions include some form of the following:
     - Don't give out personal information to strangers (such as name, address, place of work, phone number, etc.)
     - Don't fill out forms from an unknown source that request personal information.

- Be aware of possible scams; do not provide bank information or send money to strangers.
- Don't arrange to meet with or go to meet with people you don't know.
- Don't share photos that you would not want others to see.

- Review Facebook™ privacy and safety settings
  - Instruct each participant to conduct a security checkup by typing fb.me/securitycheckup into their browser and follow the instructions.

8. **Post ground rules and welcome message:**
   - Sign into the Facebook™ group.
   - Under the group title, next to your photo, under the text that reads "write something," select the photo icon. Upload the photo of the group's ground rules, developed during the in-person meeting, from your photo album. Click "done."
   - Above the photo, where it reads, "Say something about this post" type the following message: "Hi everyone! This is a photo of the ground rules we agreed upon. Let's remember and respect them throughout our discussions. 😊"
   - Select "post" to post the photo and message.

SMART Connections

SESSIONS 1-11

SESSION 1:
UNDERSTANDING HIV

Background for the Facilitator
Review the following information before leading this session.

HIV can be difficult to discuss with anyone but talking with youth about HIV poses some additional challenges. Young people living with HIV (YLHIV) are not a homogenous group, so the messages you deliver should be tailored according to your participants' ages, sexes, emotional maturity, and their existing knowledge and experience. You will be explaining some adult concepts and complex medical issues.

Before you begin your support group, it might be helpful to review some basic information about HIV:

- Human immunodeficiency virus (HIV) infects cells of the immune system and destroys or impairs their function. HIV infection results in the progressive deterioration of the immune system, breaking down the body's ability to fend off infections and diseases. Acquired immune deficiency syndrome (AIDS) refers to the most advanced stages of HIV infection.
- HIV can be transmitted through unprotected vaginal or anal intercourse or oral sex with an infected person; transfusions of contaminated blood; and sharing contaminated needles, syringes, or other sharp instruments. It can also be transmitted between a mother and her baby during pregnancy, childbirth, and breast-feeding.

Session Goals

In this session, participants will:

- Learn the definitions of HIV and AIDS.
- Learn how HIV infection occurs.
- Find out how HIV affects the body and its immune system.

DAY 1: ICEBREAKER

❑ Step 1

Sign into the Facebook Group.

❑ Step 2

Under the group title, next to your photo, under the text that reads "write something," type the following message: "Welcome to our online group! I am looking forward to sharing with you over the next couple of weeks. What are you most looking forward to learning?"

❑ Step 3

Select "post" to post the message.

DAY 2: AT-A-GLANCE

❑ Step 1:

Sign into the Facebook Group.

❑ Step 2:

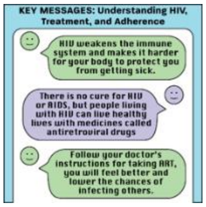

Under the group title, next to your photo, under the text that reads "write something," select the photo icon. Upload "Image #1" from the photo album on your phone. Click "done."

❑ Step 3:

Above the photo, where it reads, "Say something about this post" type the following message: "*These are some of the things that we will talk about in our first session.* 👌"

❑ Step 4:

Select "post" to post the photo and message.

DAY 3: WORD OF THE WEEK

❑ Step 1:

Sign into the Facebook Group.

Step 2:

Under the group title, next to your photo, under the text that reads “write something,” select the photo icon. Upload “Image # 2” from your photo album. Click “done.”

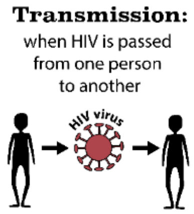

Step 3:

Above the photo, where it reads, “Say something about this post” type the following message: “Check out this week’s word of the week 👍. If you have any questions, post them here.”

Step 4:

Select “post” to post the photo and message.

DAY 4: POLL

Step 1:

Sign into the Facebook Group.

Step 2:

Under the group title, next to your photo, under the text that reads “write something,”
select the poll icon.

Step 3:

- Where you see the words “Ask something…” type the following: “HIV is spread by kissing.” Then where you see “+ Add a poll option…” type “True.” Click “Done.”
- Another “+ Add a poll option…” will appear. Type: “False.” Click “Done.”
- Select “Post” in the upper right corner.

DAY 5: DISCUSSION

Step 1:

Sign into the Facebook Group

Step 2:

Under the group title, next to your photo, under the text that reads “write something,” type the following message: “What do you know about HIV?” with you over the next couple of weeks. What are you most looking forward to learning?”

☐ Step 3:

Select “post” to post the message.

Note to Facilitator

As with other sessions, if you notice that certain group members have not yet participated this week, you might want to reach out to them privately to ask if everything is okay and to offer any support or advice they might need.

DAY 6: KEY MESSAGES

☐ Step 1:

Sign into the Facebook Group

☐ Step 2:

Under the group title, next to your photo, under the text that reads “write something,” select the photo icon. Upload Images number 3-7 from your photo album. Make sure that the “+Album” option is selected.

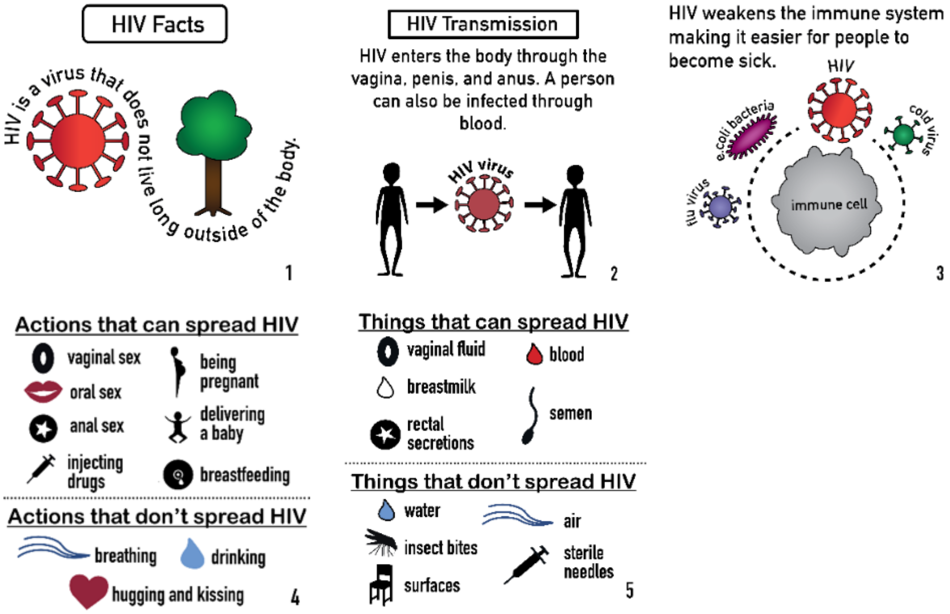

☐ Step 3:

Above the photo, where it reads, “Say something about this post” type the following message: “Do you know how HIV is transmitted? We can discuss any questions or comments you have.”

❑ **Step 4:**

Select "post" to post the photos and message.

DAY 7: DISCUSSION

❑ **Step 1:**

Sign into the Facebook Group.

❑ **Step 2:**

Under the group title, next to your photo, under the text that reads "write something,"
type the following message: "Do you have any questions about HIV or living with it?"

Note to Facilitator

As with other sessions, if you notice that certain group members have not yet participated this week, you might want to reach out to them privately to ask if everything is okay and to offer any support or advice they might need.

DAY 8: NO CONTENT TO POST

Monitor comments from participants. Encourage conversation by responding to participant posts and asking follow-up questions.

DAY 9: SOCIAL ACTIVITY

❑ **Step 1:**

Sign into the Facebook Group

❑ **Step 2:**

Under the group title, next to your photo, under the text that reads "write something," type the following message: "List one thing you about yourself that you are proud of □." with you over the next couple of weeks. What are you most looking forward to learning?"

❑ **Step 3:**

Select "post" to post the message.

Note to Facilitator

In addition to this message, you may choose to list one thing about yourself that you are proud of as an example. Possible examples include: "I am a good friend," "I am a good dancer," Etc.

DAY 10: POLL

❑ Step 1:

Sign into the Facebook Group.

❑ Step 2:

Under the group title, next to your photo, under the text that reads "write something,"
select the poll icon.

❑ Step 3:

- Where you see the words "Ask something…" type the following: "Which of the following does NOT transmit HIV?" Then where you see "+ Add a poll option…" type "Through sex." Click "Done."
- Another "+ Add a poll option…" will appear. Type: "From mother to baby through breast milk" Click "Done."
- Another "+ Add a poll option…" will appear. Type: "From mother to baby when the mum is pregnant." Click "Done."
- Another "+ Add a poll option…" will appear. Type: "Insect/mosquito." Click "Done."
- Select "Post" in the upper right corner.

DAY 11: POLL ANSWER

❑ Step 1:

Sign into the Facebook Group.

❑ Step 2:

Under the poll you created yesterday, on the bottom right hand side,
click on the word "comment."

❑ Step 3:

Where you see the words "Write a comment…" type the following: "HIV is found in blood, semen, vaginal fluid and breastmilk; HIV is not transmitted through insect bites" Click the arrow icon to post your comment.

DAY 12: DISCUSSION

- [ ] **Step 1:**

Sign into the Facebook Group.

- [ ] **Step 2:**

Under the group title, next to your photo, under the text that reads "write something," type the following message: "Is there something you have been thinking about that you would like to share with the group? 🤔"

Note to Facilitator

As with other sessions, if you notice that certain group members have not yet participated this week, you might want to reach out to them privately to ask if everything is okay and to offer any support or advice they might need.

DAY 13: NO CONTENT TO POST

Monitor comments from participants. Encourage conversation by responding to participant posts and asking follow-up questions.

DAY 14: SESSION WRAP-UP

- [ ] **Step 1:**

Sign into the Facebook Group.

- [ ] **Step 2:**

Under the group title, next to your photo, under the text that reads "write something," type the following message: "You are not alone. I am here to support you and you can support each other 🙆. If you have any questions about this session reply to this post, or message me privately."

Note to Facilitator

As with other sessions, if you notice that certain group members have not yet participated this week, you might want to reach out to them privately to ask if everything is okay and to offer any support or advice they might need.

PREPARE TO BEGIN SESSION 2.

SESSION 2:
TREATMENT AND ADHERENCE

Background for the Facilitator
Review the following information before leading this session.

Adherence, which means taking antiretroviral therapy (ART) as prescribed - taking every dose every day—is crucial to avoiding drug resistance, remaining healthy, reducing the chances of infecting a partner, and reducing the chances of delivering a baby who is HIV positive. ART prevents HIV from reproducing and multiplying in the body. If the virus stops reproducing, then the body's immune cells can live longer and provide the body with protection from infections.

Session Goals:

In this session, participants will:

- Learn how ART keeps PLHIV healthy and stops progression of HIV infection.
- Discuss the challenges of adhering to ART and strategies to overcome them.

DAY 1: AT-A-GLANCE

❑ **Step 1:**

Sign into the Facebook Group.

❑ **Step 2:**

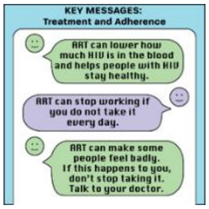

Under the group title, next to your photo, under the text that reads "write something," select the photo icon. Upload "Image #8" from your photo album. Click "done."

❑ **Step 3:**

Above the photo, where it reads, "Say something about this post" type the following message: *"Hi everyone! These are some of the things that we will talk about this session.* 👌"

❑ **Step 4:**

Select "post" to post the photo and message.

DAY 2: WORD OF THE WEEK

❑ **Step 1:**

Sign into the Facebook Group.

❑ **Step 2:**

Under the group title, next to your photo, under the text that reads "write something," select the photo icon. Upload "Image #9" from your photo album. Click "done."

Adherence:
taking ART correctly every day

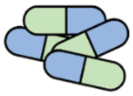

❑ **Step 3:**

Above the photo, where it reads, "Say something about this post" type the following message: "Check out this week's word of the week."

❑ **Step 4:**

Select "post" to post the photo and message.

DAY 3: DISCUSSION

❑ Step 1:

Sign into the Facebook Group.

❑ Step 2:

Under the group title, next to your photo, under the text that reads "write something," type the following message: "What does it mean to take your drugs correctly? "

Note to Facilitator

Not all drug regimens are the same. Focus on ensuring that group members understand how to take medicine exactly as directed by their health care provider. As with other sessions, if you notice that certain group members have not yet participated this week, you might want to reach out to them privately to ask if everything is okay and to offer any support or advice they might need.

DAY 4: POLL

❑ Step 1:

Sign into the Facebook Group

❑ Step 2:

Under the group title, next to your photo, under the text that reads "write something," select the poll icon.

❑ Step 3:

- Where you see the words "Ask something…" type the following: "True or false: You should only take your ART (medicine) when you feel sick? " Then where you see "+ Add a poll option…" type "True." Click "Done."
- Another "+ Add a poll option…" will appear. Type: "False." Click "Done."
- Select "Post" in the upper right corner.

DAY 5: POLL ANSWER

❑ Step 1:

Sign into the Facebook Group

❑ Step 2:

Under the poll you created yesterday, on the bottom right hand slide, click on the word "comment."

❑ Step 3:

Where you see the words “Write a comment…” type the following:
“👏 You should take your ART pills every day exactly as prescribed.” Click the arrow icon to post your comment.

DAY 6: NO CONTENT TO POST

Monitor comments from participants. Encourage conversation by responding to participant posts and asking follow-up questions.

DAY 7: ABIGAIL ADHERES TO HER ART

❑ Step 1:

Sign into the Facebook Group.

❑ Step 2:

Under the group title, next to your photo, under the text that reads “write something,” select the photo icon. Upload images number 10-16 from your photo album. Make sure that the “+Album” option is selected.

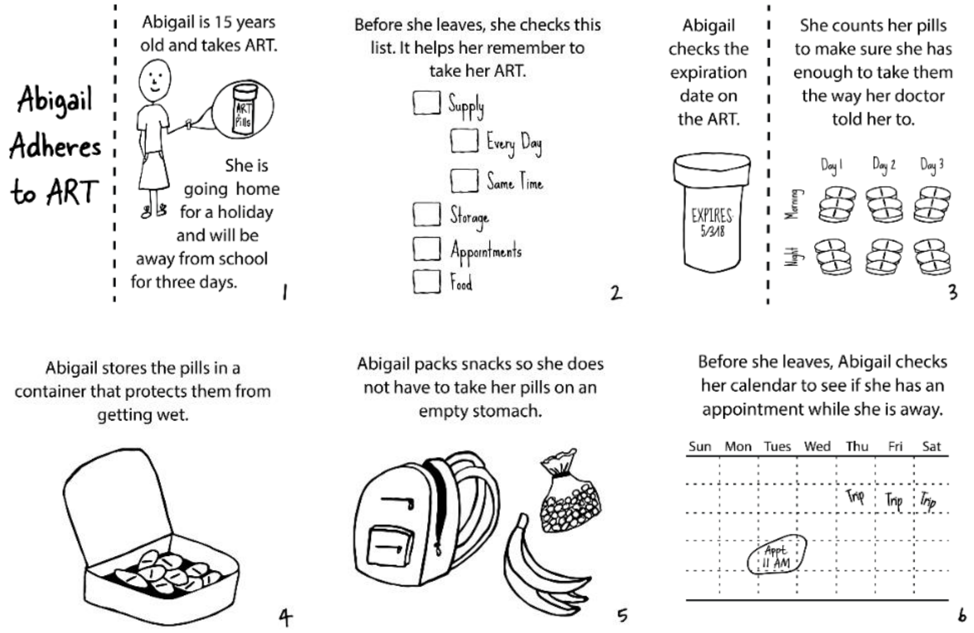

❑ Step 3:

Above the photo, where it reads, “Say something about this post” type the following message: “Click through this photo album to see Abigail’s story. Reply to this post if you have any questions or comments!”

❑ Step 4:

Select “post” to post the photos and message.

DAY 8: DISCUSSION

❑ **Step 1:**

Sign into the Facebook Group

❑ **Step 2:**

Under the group title, next to your photo, under the text that reads "write something," type the following message: "Is it ever hard for you to take your ART exactly as you are supposed to every day? Why?"

Note to Facilitator

As with other sessions, if you notice that certain group members have not yet participated this week, you might want to reach out to them privately to ask if everything is okay and to offer any support or advice they might need.

DAY 9: NO CONTENT TO POST

Monitor comments from participants. Encourage conversation by responding to participant posts and asking follow-up questions.

DAY 10: SOCIAL ACTIVITY

❑ **Step 1:**

Sign into the Facebook Group

❑ **Step 2:**

Under the group title, next to your photo, under the text that reads "write something," type the following message: "I have a riddle for you: I am something, my first letter is 'T,' I am full of 'T,' and I end with 'T.' What am I?""

Note to Facilitator

The solution to this riddle is a tea pot.

DAY 12: DISCUSSION

❑ **Step 1:**

Sign into the Facebook Group.

❑ **Step 2:**

Under the group title, next to your photo, under the text that reads "write something," select the photo icon. Upload images number 17-26 from your photo album. Make sure that the "+Album" option is selected. Type the following message: "How do you remember to take your pills every day? Do you have any tips to share with others?"

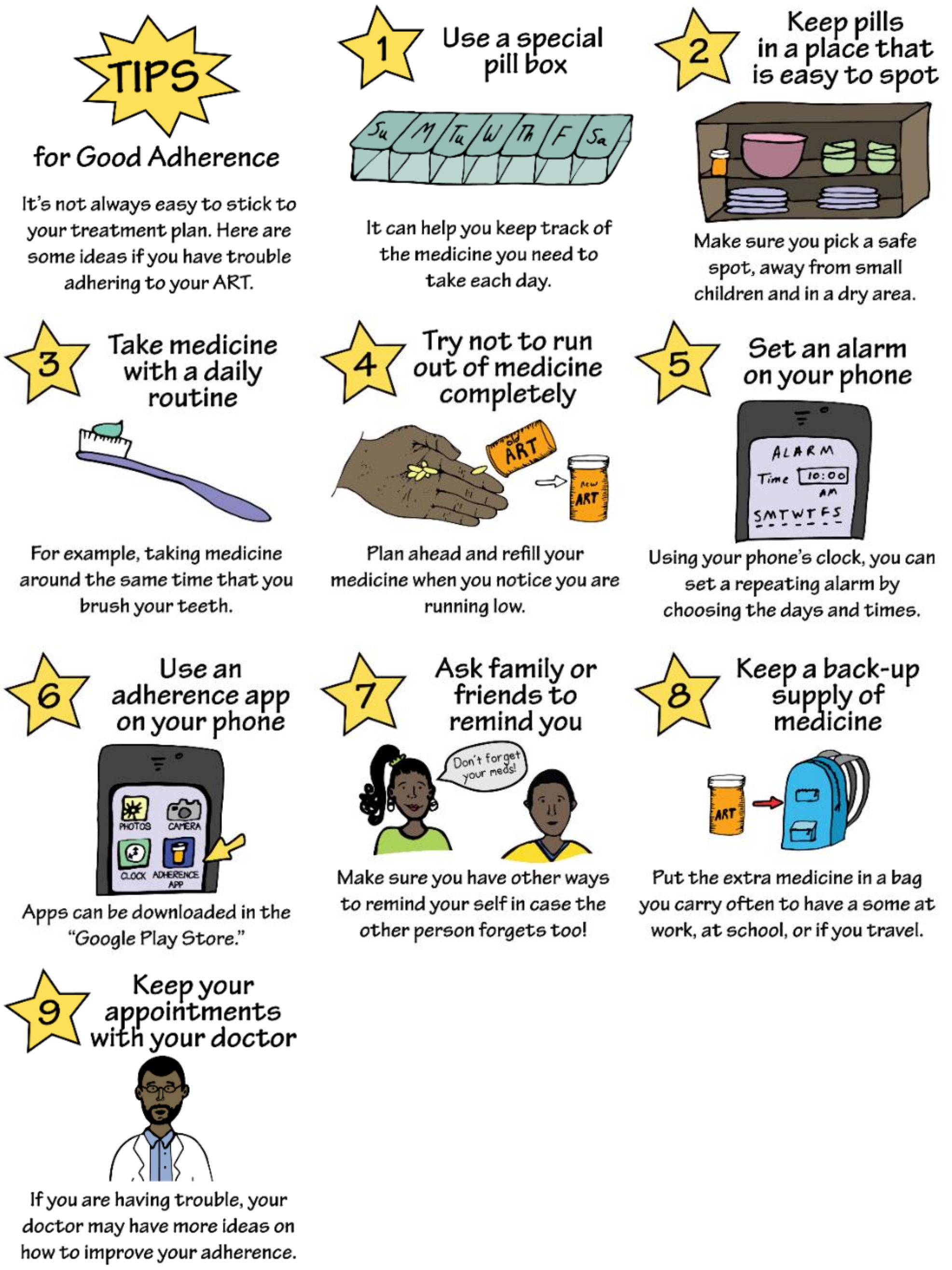

❑ **Step 3:**

Select "post" to post the photos and message.

DAY 13: NO CONTENT TO POST

Monitor comments from participants. Encourage conversation by responding to participant posts and asking follow-up questions.

DAY 14: SESSION WRAP-UP

❑ Step 1:

Sign into the Facebook Group.

❑ Step 2:

Under the group title, next to your photo, under the text that reads "write something," type the following message: "It is important to take your ART exactly as your healthcare provider says. If you are having trouble, ask for help. We can help each other. Let me know if you have any questions."

Note to Facilitator

As with other sessions, if you notice that certain group members have not yet participated this week, you might want to reach out to them privately to ask if everything is okay and to offer any support or advice they might need.

PREPARE TO BEGIN SESSION 3.

SESSION 3: DISCLOSURE

Background for the Facilitator
Review the following information before leading this session.

Disclosing one's HIV status can be beneficial but may also be risky. Family and friends can provide essential support to YLHIV if they are adequately informed. Health care providers who know their client is living with HIV can offer appropriate care and treatment. A romantic partner can provide emotional support and help practice safer sex. However, people who disclose their HIV positive status to others also face the potential of being rejected, admonished, hurt by others, or abandoned, often by the people closest to them.

Trust is an important part of disclosure. YLHIV must question: Who can I trust? If I tell someone, will he or she reject me? Will they hurt me? Will they tell someone else that I am living with HIV or have AIDS? Young people need support determining whom they can trust, deciding whom they want to tell their status, and practicing how to disclose. You can help participants in your group weigh the risks and benefits of disclosing their status to others.

Session Goals:

In this session, participants will:

- Learn the meaning of disclosure.
- Discuss the importance of trust and understand how to develop it.
- Explore the benefits and risks of disclosure.
- Provide information about safe disclosure if, and when, participants are ready to do so.

DAY 1: AT-A-GLANCE

- [ ] **Step 1:**

Sign into the Facebook Group

- [ ] **Step 2:**

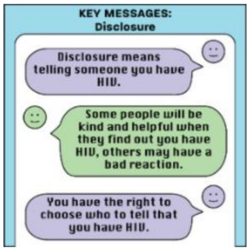

Under the group title, next to your photo, under the text that reads "write something," select the photo icon. Upload "Image #27" from your photo album. Click "done"

- [ ] **Step 3:**

Above the photo, where it reads, "Say something about this post" type the following message: "*We are starting a new session; these are some of the things that we will talk about* ✔."

- [ ] **Step 4:**

Select "post" to post the photo and message.

DAY 2: WORD OF THE WEEK

- [ ] **Step 1:**

Sign into the Facebook Group.

- [ ] **Step 2:**

Under the group title, next to your photo, under the text that reads "write something," select the photo icon. Upload "Image #28 from your photo album. Click "done."

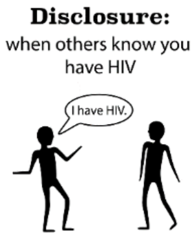

- [ ] **Step 3:**

Above the photo, where it reads, "Say something about this post" type the following message: "Check out this week's word of the week 👍."

- [ ] **Step 4:**

Select "post" to post the photo and message.

DAY 3: POLL

❑ Step 1:

Sign into the Facebook Group.

❑ Step 2:

Under the group title, next to your photo, under the text that reads "write something," select the poll icon.

❑ Step 3:

Where you see the words "Ask something…" type the following: "What is disclosure?" Then where you see "+ Add a poll option…" type "Telling someone that you have HIV." Click "Done."

- Another "+ Add a poll option…" will appear. Type: "When someone reveals that you have HIV without your permission." Click "Done."
- Another "+ Add a poll option…" will appear. Type: "Both of these." Click "Done."
- Another "+ Add a poll option…" will appear. Type: "Neither of these." Click "Done."
- Select "Post" in the upper right corner.

DAY 4: POLL ANSWER

❑ Step 1:

Sign into the Facebook Group

❑ Step 2:

Under the poll you created yesterday, on the bottom right hand slide, click on the word "comment."

❑ Step 3:

Where you see the words "Write a comment…" type the following: "Both of these are definitions of disclosure. Disclosure can be risky and/or beneficial. We will talk more about this in the coming weeks. 👏 👍" Click the arrow icon to post your comment.

DAY 5: DISCUSSION

❑ Step 1:

Sign into the Facebook Group.

Step 2:

Under the group title, next to your photo, under the text that reads "write something," type the following message: "Why can it be good to tell someone you are living with HIV? For those of you who have told someone that you are living with HIV, what was your experience like? Have you ever had an experience where someone shared your status without your permission? How did you feel?"

Note to Facilitator

If you are comfortable sharing your own experience with disclosure with the group, it may help to make participants feel more comfortable sharing theirs. As with other sessions, if you notice that certain group members have not yet participated this week, you might want to reach out to them privately to ask if everything is okay and to offer any support or advice they might need.

DAY 6: NO CONTENT TO POST

Monitor comments from participants. Encourage conversation by responding to participant posts and asking follow-up questions.

DAY 7: DISCLOSURE CARTOON

Step 1:

Sign into the Facebook Group.

Step 2:

Under the group title, next to your photo, under the text that reads "write something," select the photo icon. Upload images number 29-41 from your photo album. Make sure that the "+Album" option is selected.

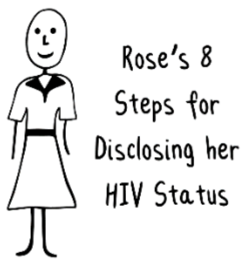

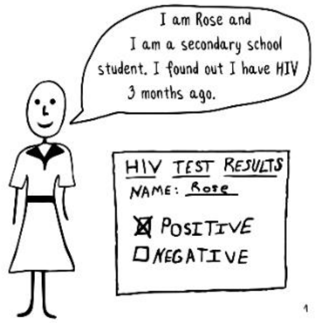

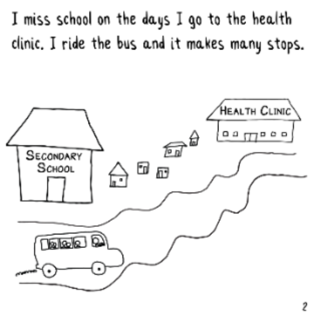

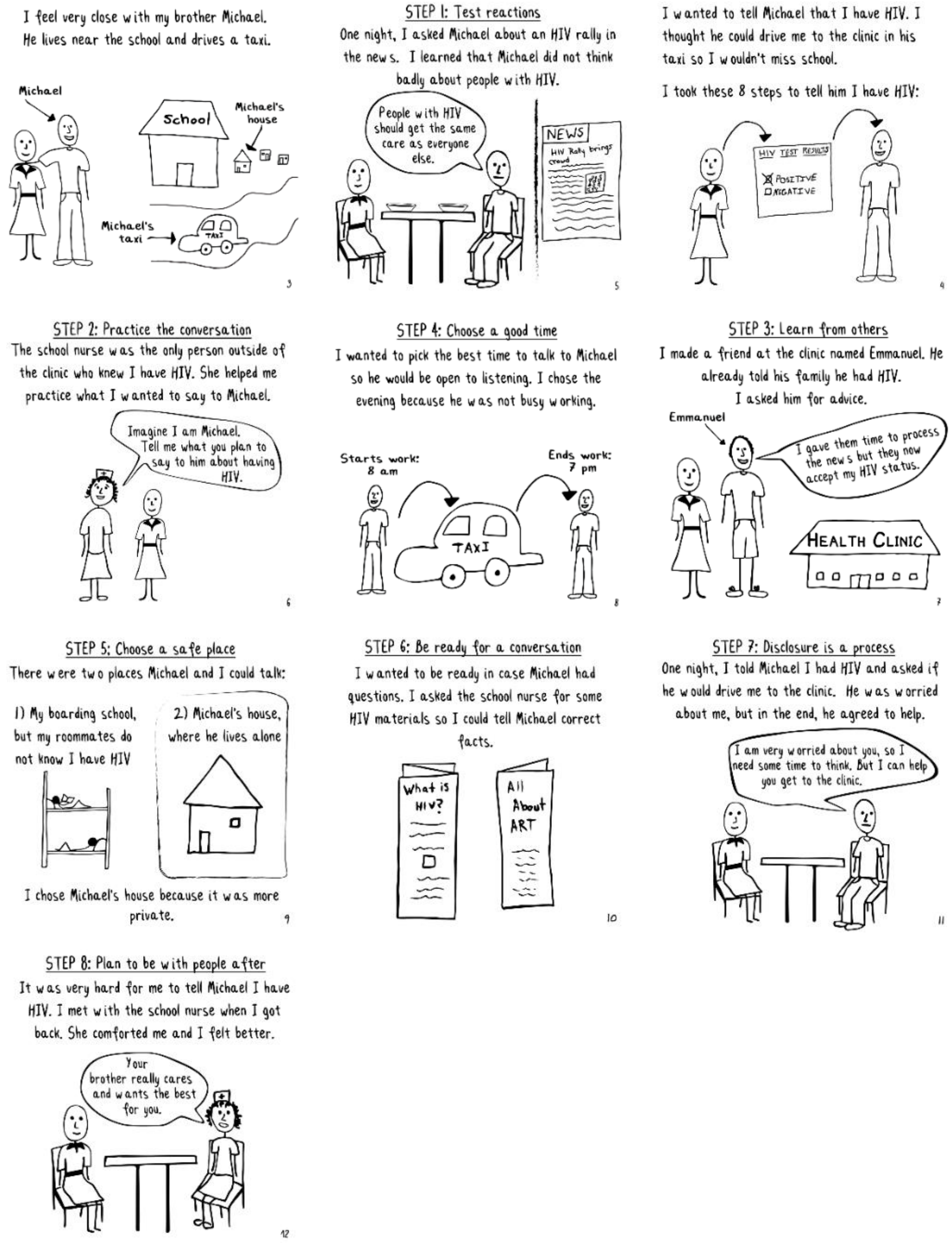

❑ Step 3:

Above the photo, where it reads, “Say something about this post” type the following message: “Check out this story about the steps Rose followed when telling someone she has HIV. What do you think about the cartoon?”

❑ Step 4:

Select “post” to post the photos and message.

DAY 8: DISCUSSION

❑ **Step 1:**

Sign into the Facebook Group.

❑ **Step 2:**

Under the group title, next to your photo, under the text that reads "write something," type the following message: "What does trust mean to you? How do you know when you can trust someone?"

Note to Facilitator

As with other sessions, if you notice that certain group members have not yet participated this week, you might want to reach out to them privately to ask if everything is okay and to offer any support or advice they might need.

DAY 9: NO CONTENT TO POST

Monitor comments from participants. Encourage conversation by responding to participant posts and asking follow-up questions.

DAY 10: SOCIAL ACTIVITY

❑ **Step 1:**

Sign into the Facebook Group.

❑ **Step 2:**

Under the group title, next to your photo, under the text that reads "write something," type the following message: "Tell us something about yourself, what is your favorite movie?"

Note to Facilitator

In addition to this message, you may choose to share your favorite movie with the group.

DAY 11: KEY MESSAGES

❑ **Step 1:**

Sign into the Facebook Group.

❑ Step 2:

Under the group title, next to your photo, under the text that reads "write something," select the photo icon. Upload images number 42-47 from your photo album. Make sure that the "+Album" option is selected.

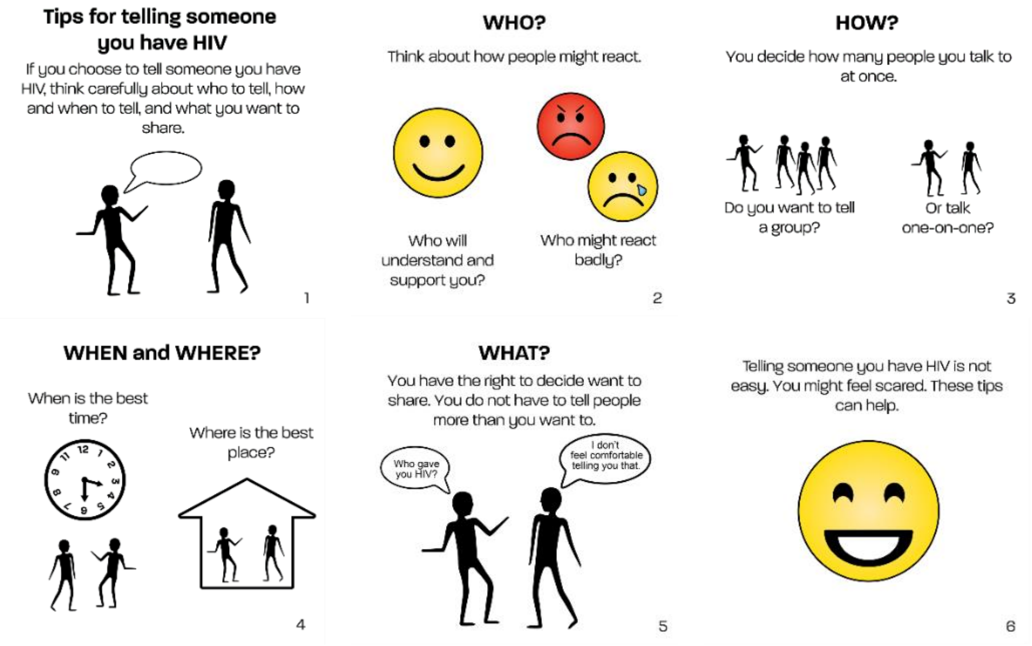

❑ Step 3:

Above the photo, where it reads, "Say something about this post" type the following message: "There can be good reasons to tell people you have HIV. Some people will be supportive and trustworthy, and others might have bad reactions. Plan your response or next steps if someone reacts very badly."

❑ Step 4:

Select "post" to post the photos and message.

DAY 12: DISCUSSION

❑ Step 1:

Sign into the Facebook Group.

❑ Step 2:

Under the group title, next to your photo, under the text that reads "write something," type the following message: "Do you have any tips about disclosure that you would like to share with the group?"

Note to Facilitator

As with other sessions, if you notice that certain group members have not yet participated this week, you might want to reach out to them privately to ask if everything is okay and to offer any support or advice they might need.

DAY 13: NO CONTENT TO POST

Monitor comments from participants. Encourage conversation by responding to participant posts and asking follow-up questions.

DAY 14: SESSION WRAP-UP

❑ **Step 1:**

Sign into the Facebook Group.

❑ **Step 2:**

Under the group title, next to your photo, under the text that reads "write something," type the following message: "Think carefully about telling people that you have HIV and remember this group is here to support you. ♡ If you have any questions about this session reply to this post, or message me privately."

Note to Facilitator

As with other sessions, if you notice that certain group members have not yet participated this week, you might want to reach out to them privately to ask if everything is okay and to offer any support or advice they might need.

PREPARE TO BEGIN SESSION 4.

SESSION 4:
EXPLORING YOUR FEELINGS

Background for the Facilitator
Review the following information before leading this session.

YLHIV may experience anxiety, depression, anger, guilt, and loss of self-esteem and confidence. They are often anxious about their future and wonder what will happen to them? Will they die? Who will care for them? How will they be treated? The effects of these different emotions can create a stress level that can become physically damaging and affect their self-care and treatment efforts.

Session Goals:

In this session, participants will:

- Identify different feelings and emotions and how people experience them.
- Discuss grief and loss and how to recognize the stages people might go through during these feelings.
- Discuss strategies for dealing with grief, anger and fear and for beginning to feel better over time.

DAY 1: AT-A-GLANCE

❑ Step 1:

Sign into the Facebook Group

❑ Step 2:

Under the group title, next to your photo, under the text that reads "write something," select the photo icon. Upload image #48 from your photo album. Click "done."

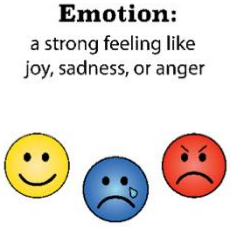

❑ Step 3:

Above the photo, where it reads, "Say something about this post" type the following message: "During this session we are going to learn to explore our feelings."

❑ Step 4:

Select "post" to post the photo and message.

DAY 2: WORD OF THE WEEK

❑ Step 1:

Sign into the Facebook Group

❑ Step 2:

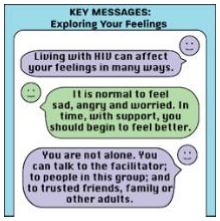

Under the group title, next to your photo, under the text that reads "write something," select the photo icon. Upload Image #49 from your photo album. Click "done"

❑ Step 3:

Above the photo, where it reads, "Say something about this post" type the following message: "Check out this week's word of the week 👍."

❑ Step 4:

Select "post" to post the photo and message.

DAY 3: DISCUSSION

Step 1:

Sign into the Facebook Group

Step 2:

Under the group title, next to your photo, under the text that reads "write something," type the following message: "What are some of the emotions that you have had over the past week? 😠🙂😢🤪"

Note to Facilitator

As with other sessions, if you notice that certain group members have not yet participated this week, you might want to reach out to them privately to ask if everything is okay and to offer any support or advice they might need.

DAY 4: NO CONTENT TO POST

Monitor comments from participants. Encourage conversation by responding to participant posts and asking follow-up questions.

DAY 5: KEY MESSAGE

❑ Step 1:

Sign into the Facebook Group

❑ Step 2:

Under the group title, next to your photo, under the text that reads "write something," select the photo icon. Upload images #50-54 from your photo album.

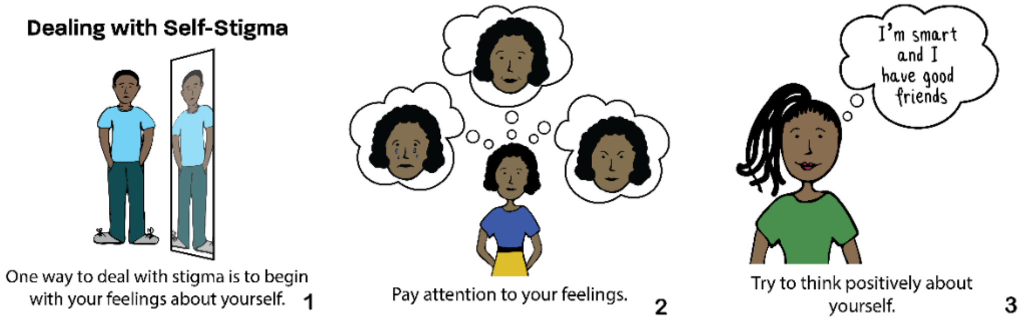

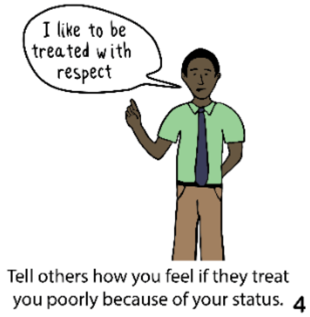

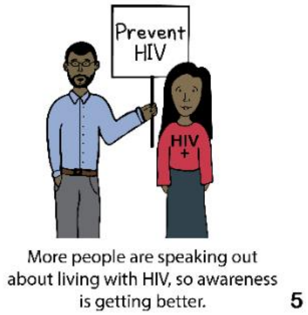

❑ Step 3:

Above the photo, where it reads, "Say something about this post" type the following message: "Do you know what self-stigma is?"

❑ Step 4:

Select "post" to post the photos and message.

DAY 6: STAGES OF ACCEPTANCE

❑ Step 1:

Sign into the Facebook Group

❑ Step 2:

Under the group title, next to your photo, under the text that reads "write something," select the photo icon. Upload images number 55-60 from your photo album.

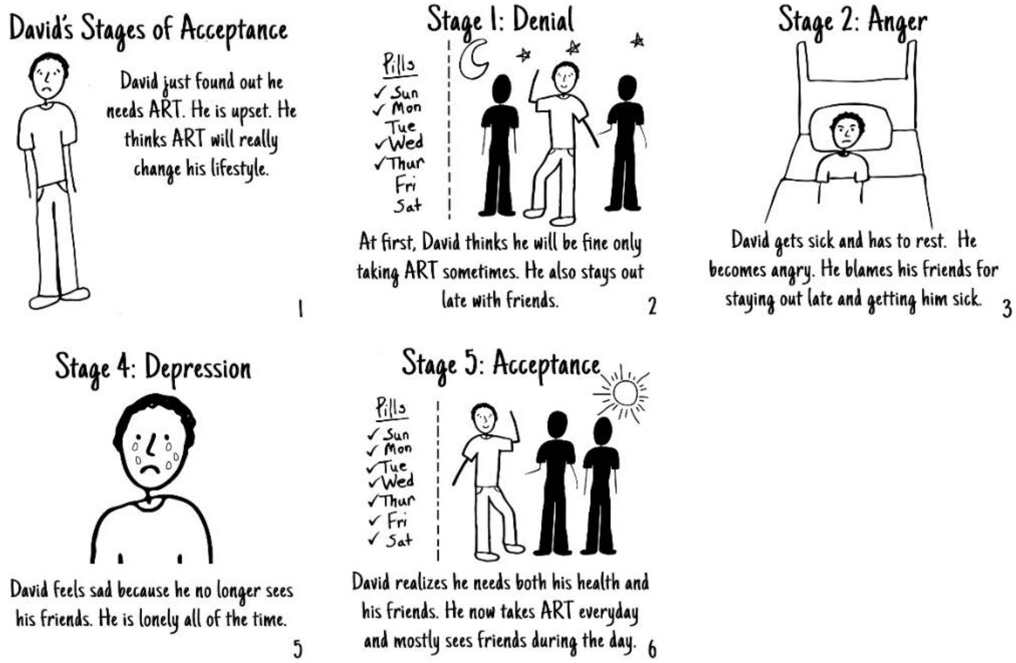

Step 3:

Above the photo, where it reads, "Say something about this post" type the following message: "Many people experience these feelings when learning that they have HIV."

Step 4:

Select "post" to post the photos and message.

DAY 7: SOCIAL ACTIVITY

Step 1:

Sign into the Facebook Group

Step 2:

Under the group title, next to your photo, under the text that reads "write something," type the following message: "I have a riddle for you: I am something, I have 24 legs but I cannot walk. What am I?"

Note to Facilitator

The solution to this riddle is a crate of mineral water.

DAY 8: WORD OF THE WEEK

Step 1:

Sign into the Facebook Group.

Step 2:

Under the group title, next to your photo, under the text that reads "write something," select the photo icon. Upload Image #61 from your photo album. Click "done."

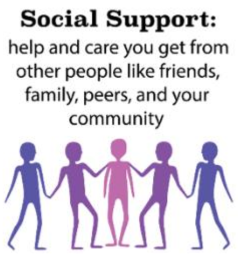

Step 3:

Above the photo, where it reads, "Say something about this post" type the following message: "We can act as each other's support network!"

Step 4:

Select "post" to post the photo and message.

DAY 9: DISCUSSION

Step 1:

Sign into the Facebook Group.

Step 2:

Under the group title, next to your photo, under the text that reads "write something," type the following message: "Who are some people you can talk to when you are feeling sad?"

Step 3:

Click on the word "Post" in the upper right corner of the screen to post the message.

Note to Facilitator

Thank participants who share for sharing their experiences and monitor the discussion closely to ensure that all participants are responding in a way that is supportive to one another. As with other sessions, if you notice that certain group members have not yet participated this week, you might want to reach out to them privately to ask if everything is okay and to offer any support or advice they might need.

DAY 10: NO CONTENT TO POST

Monitor comments from participants. Encourage conversation by responding to participant posts and asking follow-up questions.

DAY 11: POLL

Step 1:

Sign into the Facebook Group.

Step 2:

Under the group title, next to your photo, under the text that reads "write something," select the poll icon.

❑ Step 3:

- Where you see the words “Ask something…” type the following: “What can you do if you are feeling upset?” Then where you see “+ Add a poll option…” type “Try to calm down.” Click “Done.”
- Another “+ Add a poll option…” will appear. Type: “Talk to a supportive friend or adult.” Click “Done.”
  Another “+ Add a poll option…” will appear. Type: “Do something you enjoy.”
  Another “+ Add a poll option…” will appear. Type: “Any of these.” Click “Done.”
- Select “Post” in the upper right corner.

DAY 12: POLL ANSWER

❑ Step 1:

Sign into the Facebook Group

❑ Step 2:

Under the poll you created yesterday, on the bottom right hand side, click on the word “comment.”

❑ Step 3:

Where you see the words “Write a comment…” type the following: “The correct answer to this poll is “any of these.” It is normal to experience feelings about living with HIV or about life in general. None of you are alone, we can all support each other 🤗.” Click the arrow icon to post your comment.

DAY 13: RELIGION STORY

❑ Step 1:

Sign into the Facebook Group

❑ Step 2:

Under the group title, next to your photo, text that reads “write something,” select the photo icon. Upload “Images number 62-71 from your photo album. Make sure that the “+Album” option is selected.

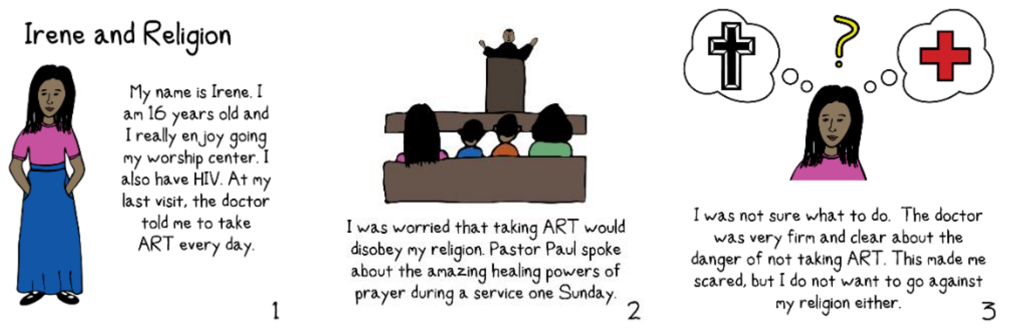

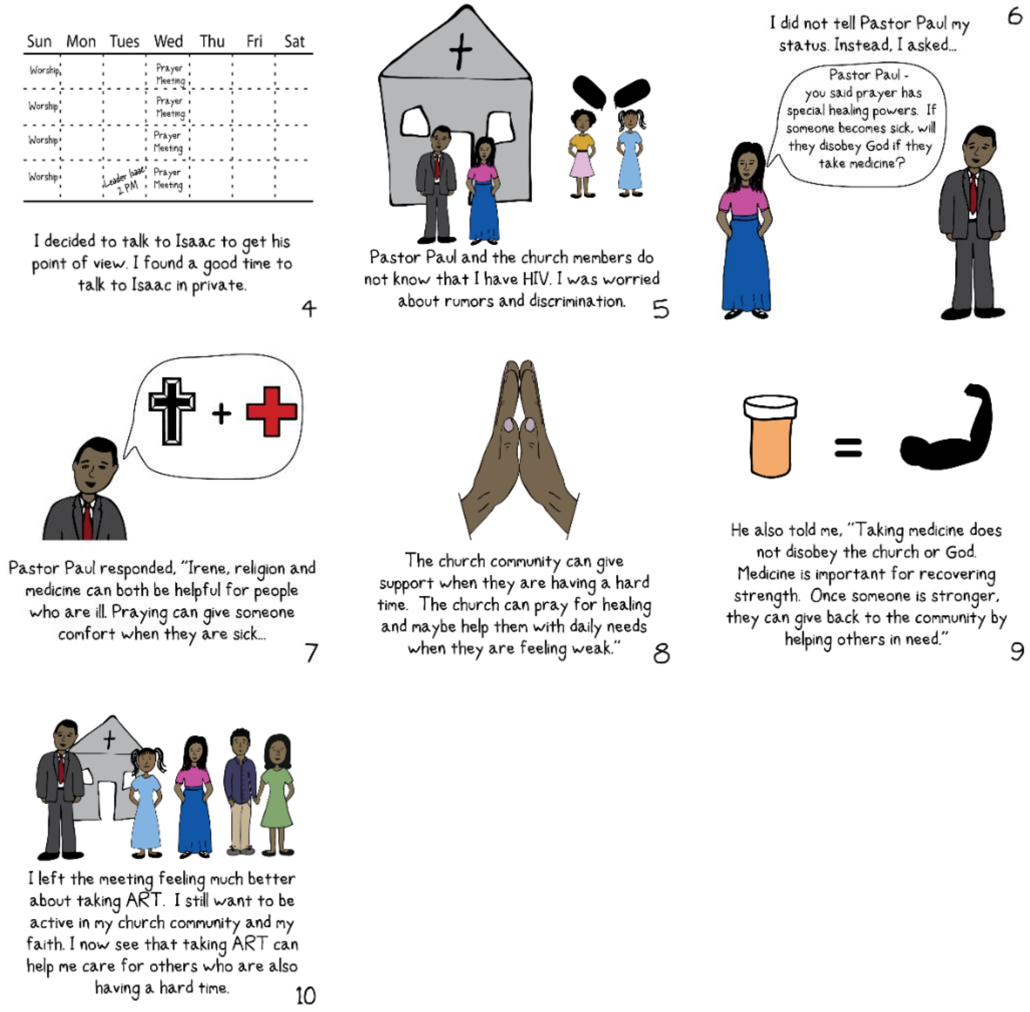

❑ Step 3:

Above the photo, where it reads, "Say something about this post" type the following message: "There are many places in your community that offer social support; some people find support from religious groups. This story is about a young woman named Irene with questions about ARTs and her religion."

❑ Step 4:

Select "post" to post the photos and message.

DAY 14: WRAP-UP

❑ Step 1:

Sign into the Facebook Group

❑ Step 2:

Under the group title, next to your photo, under the text that reads "write something," type the following message: "Thank you for your contributions this week! In the coming weeks, remember the strategies for managing emotions we discussed during this session."

Note to Facilitator

As with other sessions, if you notice that certain group members have not yet participated this week, you might want to reach out to them privately to ask if everything is okay and to offer any support or advice they might need.

PREPARE TO BEGIN SESSION 5.

SESSION 5:
NUTRITION AND HEALTH

Background for the Facilitator
Review the following information before leading this session.

Good nutrition and healthy habits are important for everyone. For YLHIV, good nutrition can help to reduce the side-effects of ART, improve response to treatment, and improve quality of life. HIV can interfere with the body's ability to absorb important nutrients. People living with HIV can get infections called opportunistic infections or OIs. OIs can cause symptoms such as diarrhea and vomiting, that can prevent the body from absorbing vitamins, minerals and other nutrients it needs to stay healthy. It is especially important for YLHIV to maintain good nutrition and get regular physical exercise to help maintain their health and prevent the progression of HIV to AIDS.

Session Goals:

In this session, participants will:

- Learn how nutrition affects HIV and what good nutrition is.
- Learn how drugs and alcohol affect HIV.
- Consider strategies for healthy living.

DAY 1: AT-A-GLANCE

❑ Step 1:

Sign into the Facebook Group

❑ Step 2:

Under the group title, next to your photo, under the text that reads "write something," select the photo icon. Upload "Image #72" from your photo album. Click "done"

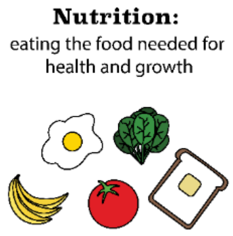

❑ Step 3:

Above the photo, where it reads, "Say something about this post" type the following message: *"Hi everyone! These are some of the things that we will talk about this session* ."

❑ Step 4:

Select "post" to post the photo and message.

DAY 2: WORD OF THE WEEK

❑ Step 1:

Sign into the Facebook Group.

❑ Step 2:

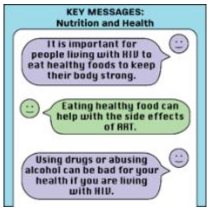

Under the group title, next to your photo, under the text that reads "write something," select the photo icon. Upload "Image #73" from your photo album. Click "done"

❑ Step 3:

Above the photo, where it reads, "Say something about this post" type the following message: "Check out this week's word of the week."

❑ Step 4:

Select "post" to post the photo and message.

DAY 3: POLL

❑ Step 1:

Sign into the Facebook Group.

❑ Step 2:

Under the group title, next to your photo, under the text that reads “write something,” select the poll icon.

❑ Step 3:

- Where you see the words “Ask something…” type the following: “Which of the following are part of a healthy diet?” Then where you see “+ Add a poll option…” type “Plenty of fruits and vegetables to provide fiber, vitamins, and minerals.” Click “Done.”
- Another “+ Add a poll option…” will appear. Type: “StarchyStarchy carbohydrates to give you energy—like whole-meal, pasta, and bread.” Click “Done.”
- Another “+ Add a poll option…” will appear. Type: “Some dairy—like milk or yogurt.” Click “Done.”
- Another “+ Add a poll option…” will appear. Type: “Some protein—like lean meat, fish, eggs, and beans.” Click “Done.”
- Another “+ Add a poll option…” will appear. Type: “Small amounts of fats and sugars.” Click “Done.”
- Another “+ Add a poll option…” will appear. Type: “All of the above” Click “Done.”
- Select “Post” in the upper right corner.

DAY 4: POLL ANSWER

❑ Step 1:

Sign into the Facebook Group

❑ Step 2:

Under the poll you created yesterday, on the bottom right hand side, click on the word “comment.”

❑ Step 3:

Where you see the words “Write a comment…” type the following: “The correct response is all of the above! 🎉 👏.” Click the arrow icon to post your comment.

DAY 5: KEY MESSAGES

❑ Step 1:

Sign into the Facebook Group

Step 2:

Under the group title, next to your photo, under the text that reads "write something," select the photo icon. Upload images number 74-80 from your photo album. Make sure that the "+Album" option is selected.

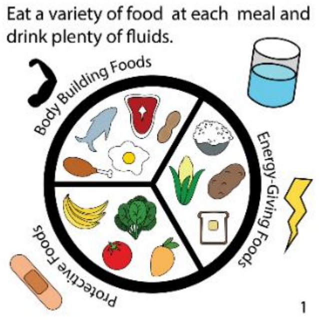

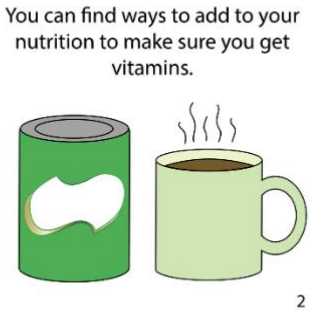

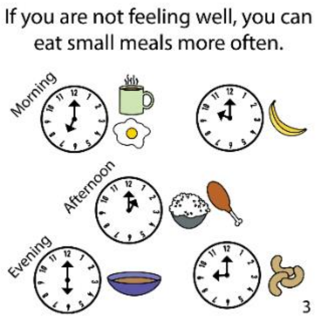

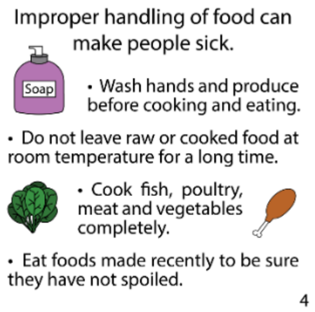

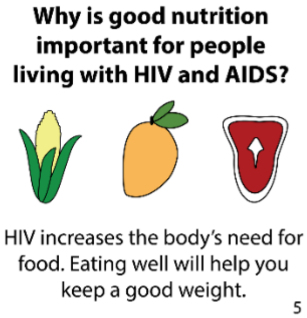

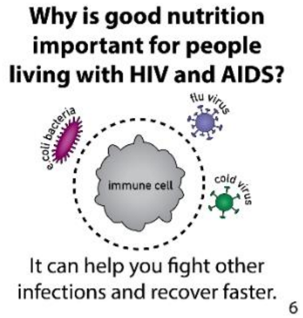

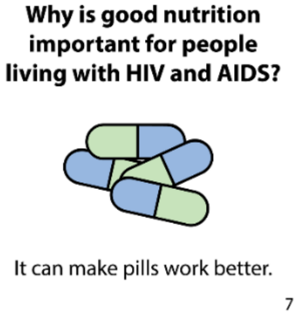

Step 3:

Above the photo, where it reads, "Say something about this post" type the following message: "Here is some more information about nutrition and health ."

Step 4:

Select "post" to post the photos and message.

DAY 6: DISCUSSION

Step 1:

Sign into the Facebook Group

❑ **Step 2:**

Under the group title, next to your photo, under the text that reads "write something," type the following message: "What are some ways you can improve the foods you eat to stay healthy? 💪"

Note to Facilitator

As with other sessions, if you notice that certain group members have not yet participated this week, you might want to reach out to them privately to ask if everything is okay and to offer any support or advice they might need.

DAY 7: NO CONTENT TO POST

Monitor comments from participants. Encourage conversation by responding to participant posts and asking follow-up questions.

DAY 8: SOCIAL ACTIVITY

❑ **Step 1:**

Sign into the Facebook Group

❑ **Step 2:**

Under the group title, next to your photo, under the text that reads "write something," type the following message: "It is Selfie day! It's selfie day! Post a 'things on point' selfie 👌 📱 ☝"

Note to Facilitator

If you choose to post your own "things on point selfie," select the photo icon, upload your selfie and type the message above in the "say something about this post" box.

DAY 9: DISCUSSION

❑ **Step 1:**

Sign into the Facebook Group

❑ **Step 2:**

Under the group title, next to your photo, under the text that reads "write something," type the following message: "Good hygiene is very important when you

are living with HIV because it helps to prevent infections. What can you do every day to stay clean and prevent germs from getting into your body?"

DAY 10: NO CONTENT TO POST

Monitor comments from participants. Encourage conversation by responding to participant posts and asking follow-up questions.

Note to Facilitator

As with other sessions, if you notice that certain group members have not yet participated this week, you might want to reach out to them privately to ask if everything is okay and to offer any support or advice they might need.

DAY 11: ADAM'S STORY

❑ Step 1:

Sign into the Facebook Group

❑ Step 2:

Under the group title, next to your photo, under the text that reads "write something," select the photo icon. Upload images number 81-88 from your photo album. Make sure that the "+Album" option is selected.

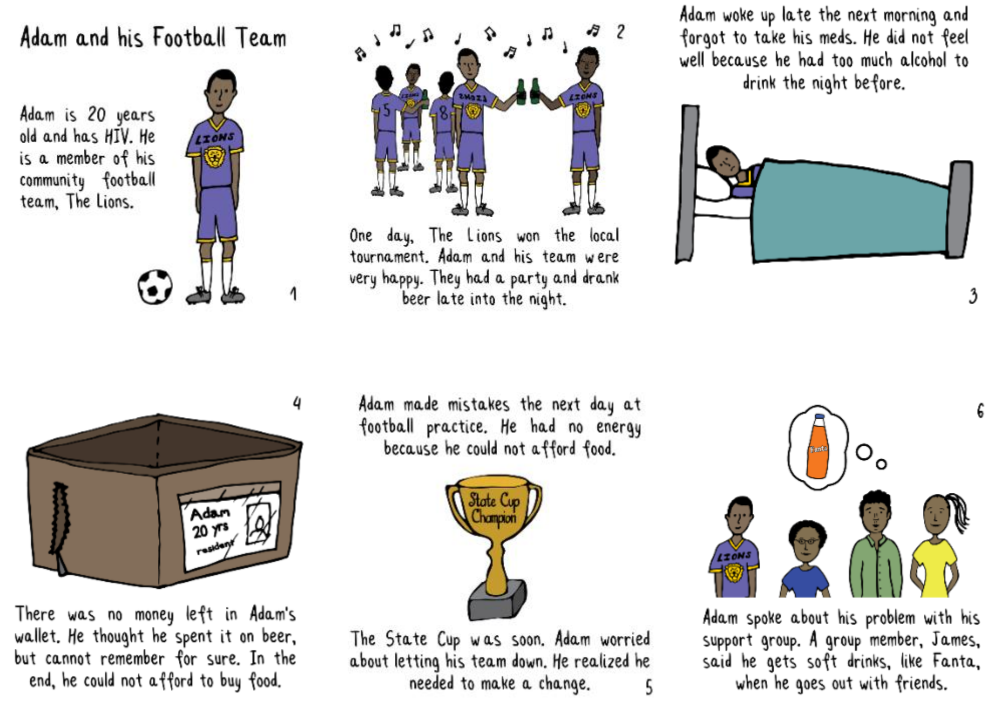

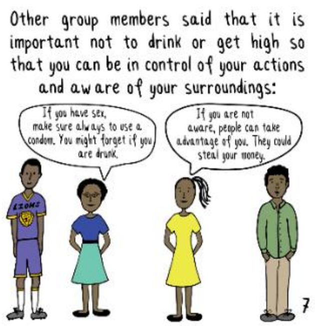

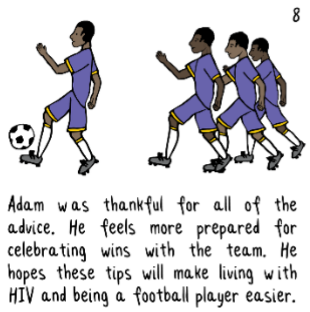

❑ Step 3:

Above the photo, where it reads, “Say something about this post” type the following message: “Another way to live a healthy lifestyle is to limit alcohol use and avoid drugs, check out Adam’s story to learn more.”

❑ Step 4:

Select “post” to post the photos and message.

DAY 12: POLL

❑ Step 1:

Sign into the Facebook Group

❑ Step 2:

Under the group title, next to your photo, under the text that reads “write something,”
select the poll icon.

❑ Step 3:

- Where you see the words “Ask something…” type the following:
  “Why is it important to avoid drugs and excessive alcohol use when living with HIV?” Then where you see “+ Add a poll option…” type
  “They can interfere with ART.” Click “Done.”
- Another “+ Add a poll option…” will appear. Type:
  “They can cause you to make poor decisions.” Click “Done.”
- Another “+ Add a poll option…” will appear. Type:
  “They weaken the immune system making it more likely to get other infections.” Click “Done.”
- Another “+ Add a poll option…” will appear. Type:
  “All of the above.” Click “Done.” Select “Post” in the upper right corner.

DAY 13: POLL ANSWER

❑ Step 1:

Sign into the Facebook Group.

❑ **Step 2:**

Under the poll you created yesterday, on the bottom right hand slide, click on the word "comment."

❑ **Step 3:**

Where you see the words "Write a comment…" type the following: "Nice work! The correct response is all of the above!" Click the arrow icon to post your comment.

DAY 14: SESSION WRAP-UP

❑ **Step 1:**

Sign into the Facebook Group

❑ **Step 2:**

Under the group title, next to your photo, under the text that reads "write something," type the following message: "It is important to stay healthy, but it's not always easy. There are people who can help. If you have any questions about this session reply to this post, or message me privately."

Note to Facilitator

As with other sessions, if you notice that certain group members have not yet participated this week, you might want to reach out to them privately to ask if everything is okay and to offer any support or advice they might need.

PREPARE TO BEGIN SESSION 6.

SESSION 6:
REPRODUCTIVE HEALTH

Background for the Facilitator
Review the following information before leading this session.

Part of youth is the exciting and sometimes awkward phase of starting to date and have relationships. YLHIV can be faced with conflicting emotions—they may want to have a girlfriend or boyfriend, but they may feel their sexuality and their relationships are redefined because they are living with HIV. Some YLHIV have become infected through sexual activity and may have complicated emotions about relationships and sexuality. While YLHIV need to consider how living with HIV plays a role in their relationships, they also need to know that they can have relationships and have the right to do so.

- PLHIV can have romantic and sexual relationships, get married and have healthy children.
- Sexual and reproductive health rights are recognized around the world as human rights. Along with these rights comes great responsibility, particularly for those living with HIV.
- Within relationships, everyone has the right to decide if and when to have sex, and to say no if he/she is not ready.
- When youth decide to have a sexual relationship, including getting married, it is important to disclose their HIV status to their partner and to know their partner's HIV status before having sex.
- Couples can take measures to protect each other from transmitting HIV to each other (more in next section on positive prevention).
- Although there are very effective methods to prevent unintended pregnancy, no method is 100% effective and when people decide to have sex, they must consider the possibility of getting pregnant and having a baby.

Session Goals:

In this session, participants will:

- Learn what sexual rights are and how they relate to living with HIV.
- Discuss how to make decisions about relationship and sex.

DAY 1: AT-A-GLANCE

- [ ] **Step 1:**

Sign into the Facebook Group.

- [ ] **Step 2:**

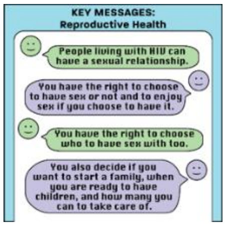

Under the group title, next to your photo, under the text that reads “write something,” select the photo icon. Upload “Image #90 from your photo album. Click “done.”

- [ ] **Step 3:**

Above the photo, where it reads, “Say something about this post” type the following message: *“This session is all about Reproductive Health.”*

- [ ] **Step 4:**

Select “post” to post the photo and message.

DAY 2: WORD OF THE WEEK

- [ ] **Step 1:**

Sign into the Facebook Group.

- [ ] **Step 2:**

Under the group title, next to your photo, under the text that reads “write something,” select the photo icon. Upload “Image #91 from your photo album. Click “done.”

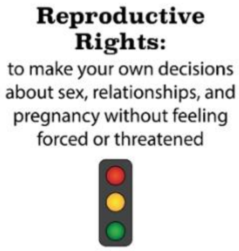

- [ ] **Step 3:**

Above the photo, where it reads, “Say something about this post” type the following message: “Check out this week’s word of the week 👍.”

- [ ] **Step 4:**

Select “post” to post the photo and message.

DAY 3: POLL

❑ **Step 1:**

Sign into the Facebook Group.

❑ **Step 2:**

Under the group title, next to your photo, under the text that reads "write something," select the poll icon.

❑ **Step 3:**

- Where you see the words "Ask something…" type the following: "You have the right to decide when you are ready to have sex"
- Then where you see "+ Add a poll option…" type "True." Click "Done."
- Another "+ Add a poll option…" will appear. Type: "False." Click "Done." Select "Post" in the upper right corner.

DAY 4: POLL ANSWER

❑ **Step 1:**

Sign into the Facebook Group.

❑ **Step 2:**

Under the poll you created yesterday, on the bottom right hand side, click on the word "comment."

❑ **Step 3:**

Where you see the words "Write a comment…" type the following: "The correct answer to this poll is true, you have the right to decide when you are ready for sex and you ***ALWAYS*** have the right to refuse sex." Click the arrow icon to post your comment.

DAY 5: SEXUAL RIGHTS

❑ **Step 1:**

Sign into the Facebook Group.

❑ **Step 2:**

Under the group title, next to your photo, under the text that reads "write something," select the photo icon. Upload images number 92- from your photo album. Make sure that the "+Album" option is selected.

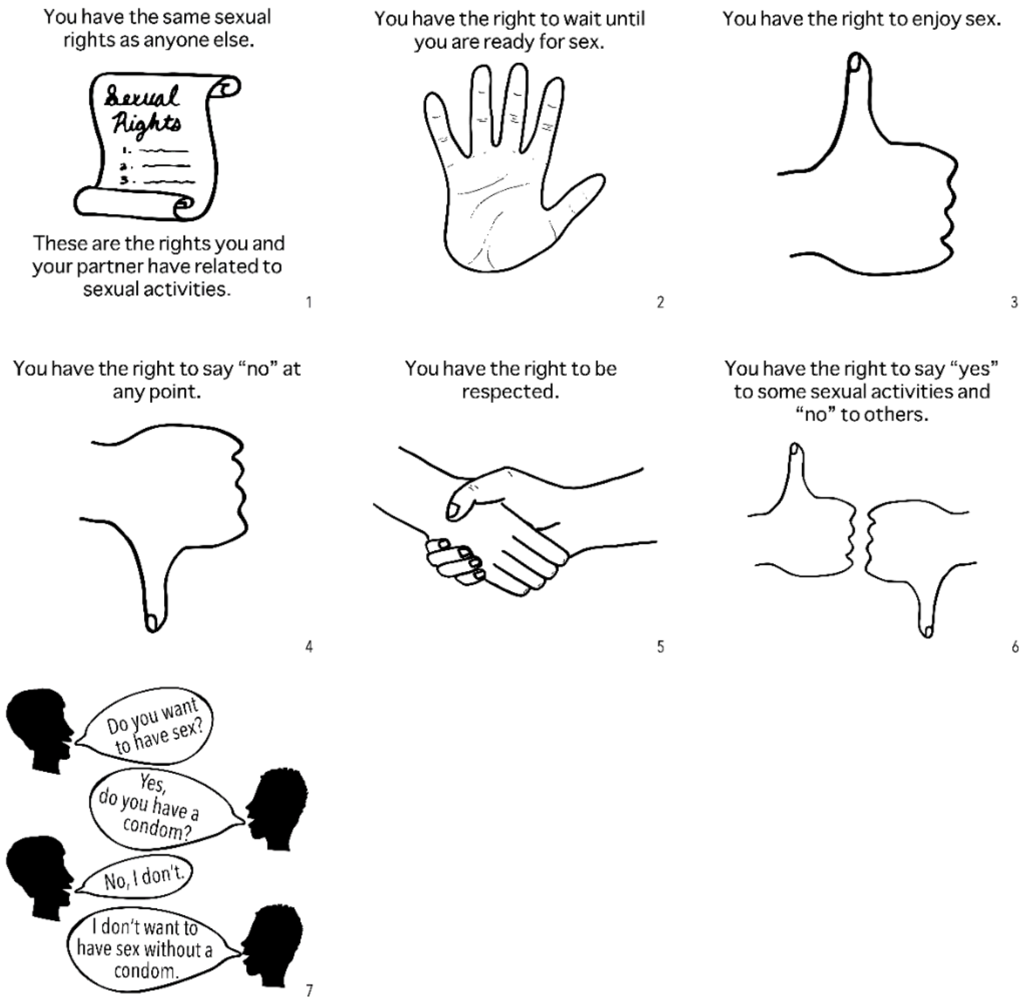

❑ Step 3:

Above the photo, where it reads, “Say something about this post” type the following message: “These are sexual rights; everyone has these rights.”

❑ Step 4:

Select “post” to post the photos and message.

DAY 6: SOCIAL ACTIVITY

❑ Step 1:

Sign into the Facebook Group.

❑ Step 2:

Under the group title, next to your photo, under the text that reads “write something,” type the following message: “What is your favorite song ♫?”

Note to Facilitator

In addition to this message, you may choose to list your favorite song.

DAY 7: DISCUSSION

- [ ] **Step 1:**

Sign into the Facebook Group

- [ ] **Step 2:**

Under the group title, next to your photo, under the text that reads "write something," type the following message: "At some point in your life, you might decide that you want to start a family. This is completely normal. How will you know you are ready to have sex? How does living with HIV affect your decision?"

Note to Facilitator

As with other sessions, if you notice that certain group members have not yet participated this week, you might want to reach out to them privately to ask if everything is okay and to offer any support or advice they might need.

DAY 8: NO CONTENT TO POST

Monitor comments from participants. Encourage conversation by responding to participant posts and asking follow-up questions.

DAY 9: KEY MESSAGES

- [ ] **Step 1:**

Sign into the Facebook Group.

- [ ] **Step 2:**

Under the group title, next to your photo, under the text that reads "write something," select the photo icon. Upload images number 99-107 from your photo album. Make sure that the "+Album" option is selected.

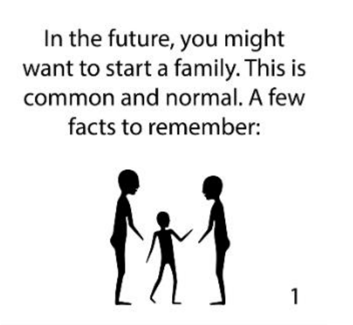

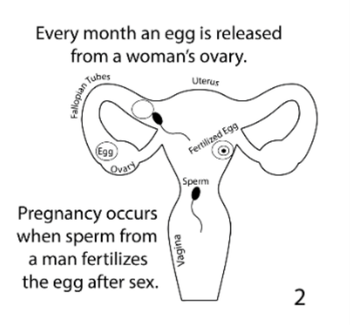

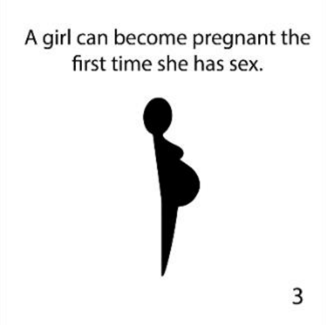

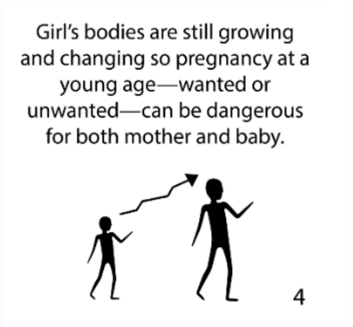

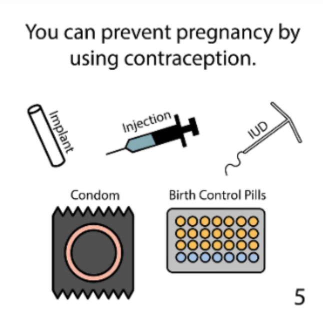

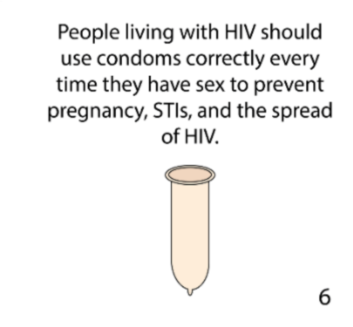

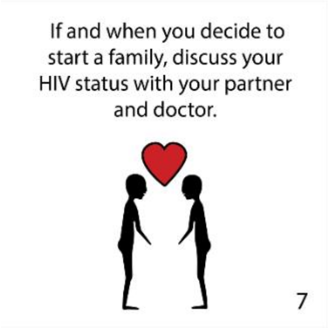

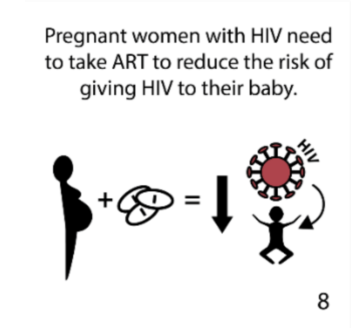

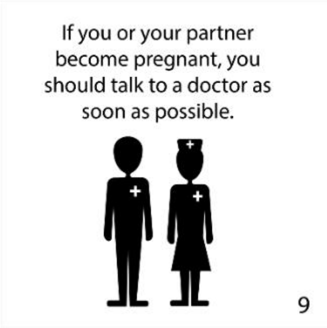

❑ **Step 3:**

Above the photo, where it reads, "Say something about this post" type the following message: "Here is some information about pregnancy prevention and planning . Post your comments and questions; we can discuss them together!"

❑ **Step 4:**

Select "post" to post the photos and message.

DAY 10: POLL

❑ **Step 1:**

Sign into the Facebook Group.

❑ **Step 2:**

Under the group title, next to your photo, under the text that reads "write something," select the poll icon.

❑ **Step 3:**

- Where you see the words "Ask something…" type the following: "By taking ART, a mum can dramatically reduce the risk of transmitting HIV to her baby during pregnancy, birth, and breastfeeding."
- Then where you see "+ Add a poll option…" type "True." Click "Done."
- Another "+ Add a poll option…" will appear. Type: "False." Click "Done." Select "Post" in the upper right corner.

DAY 11: POLL ANSWER

❑ **Step 1:**

Sign into the Facebook Group.

❑ **Step 2:**

Under the poll you created yesterday, on the bottom right hand slide, click on the word "comment."

❑ **Step 3:**

Where you see the words "Write a comment…" type the following: "The correct answer to this poll is true. Women with HIV who are taking ART might become pregnant. If this happens to you, you should talk with your doctor or healthcare provider to discuss the risks and benefits of the drugs to the baby and whether the medicines need to be changed." Click the arrow icon to post your comment.

DAY 12

❑ **Step 1:**

Sign into the Facebook Group

❑ **Step 2:**

Under the group title, next to your photo, under the text that reads "write something," type the following: http://www.myquestion.org/ An image that says, "Learning about Living in Nigeria," should appear.

❑ **Step 3:**

Next to the website link, type the following message: "This website provides answers to questions about relationships and sexual health as well as health services in Nigeria."

❑ **Step 4:**

Click on the word "Post" in the upper right corner of the screen to post the website and message.

DAY 13: SOCIAL ACTIVITY

❑ **Step 1:**

Sign into the Facebook Group

❑ **Step 2:**

Under the group title, next to your photo, under the text that reads "write something," type the following message: "I have a riddle for you: I am something, I am the longest rope in the world. What am I?"

Note to Facilitator

The solution to this riddle is rain.

DAY 14: WRAP-UP

❑ **Step 1:**

Sign into the Facebook Group

❑ **Step 2:**

Under the group title, next to your photo, under the text that reads "write something," type the following message: "I know that this information can cause people to feel shy, but if you have any questions about this session please feel free to reply to this post, or message me privately. There is nothing to be embarrassed about."

Note to Facilitator

As with other sessions, if you notice that certain group members have not yet participated this week, you might want to reach out to them privately to ask if everything is okay and to offer any support or advice they might need.

PREPARE TO BEGIN SESSION 7.

SESSION 7:
POSITIVE HEALTH, DIGNITY, AND PREVENTION

Background for the Facilitator
Review the following information before leading this session.

Incorporating prevention methods into one's life can be difficult, whether you are living with HIV or not. YLHIV may experience unique challenges when trying to practice positive prevention. These may include stigma and discrimination, lack of accurate information and lack of access to care and prevention services. While knowing their status and gathering information is a start, it helps if the group members can explore their personal roles in preventing the spread of HIV and STIs.

As a facilitator, you are not expected to know all the answers. If someone asks a difficult question, reach out to a health care provider to get more information before responding and/or direct participants to other resources in your area, including the nearest health care facility. It is always ok to tell participants that you will find a response to their question and follow-up.

Session Goals:

In this session, participants will:

- Learn the meaning of positive prevention.
- Discuss strategies for positive prevention.

DAY 1: AT-A-GLANCE

❑ Step 1:

Sign into the Facebook Group.

❑ Step 2:

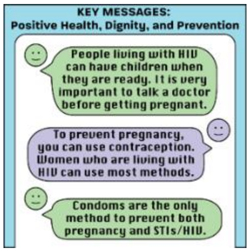

Under the group title, next to your photo, under the text that reads "write something," select the photo icon. Upload "Image #108" from your photo album. Click "done"

❑ Step 3:

Above the photo, where it reads, "Say something about this post" type the following message: *"We are starting a new session; these are some of the things that we will talk about* 👌."

❑ Step 4:

Select "post" to post the photo and message.

DAY 2: WORD OF THE WEEK

❑ Step 1:

Sign into the Facebook Group.

❑ Step 2:

Under the group title, next to your photo, under the text that reads "write something," select the photo icon. Upload "Image #109" from your photo album. Click "done"

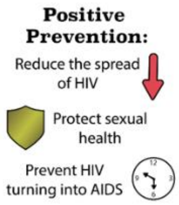

❑ Step 3:

Above the photo, where it reads, "Say something about this post" type the following message: "Check out this week's word of the week 📅."

❑ Step 4:

Select "post" to post the photo and message.

DAY 3: DISCUSSION

❑ Step 1:

Sign into the Facebook Group

❑ Step 2:

Under the group title, next to your photo, under the text that reads "write something,"
type the following message: "What does positive prevention mean to you?"

Note to Facilitator

As with other sessions, if you notice that certain group members have not yet participated this week, you might want to reach out to them privately to ask if everything is okay and to offer any support or advice they might need.

DAY 4: NO CONTENT TO POST

Monitor comments from participants. Encourage conversation by responding to participant posts and asking follow-up questions.

DAY 5: ALEX'S STORY

❑ Step 1:

Sign into the Facebook Group

❑ Step 2:

Under the group title, next to your photo, under the text that reads "write something," select the photo icon. Upload images #110-115 from your photo album. Make sure that the "+Album" option is selected.

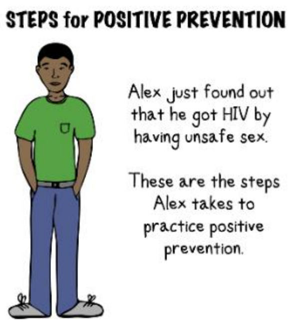

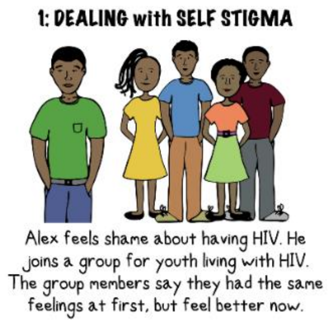

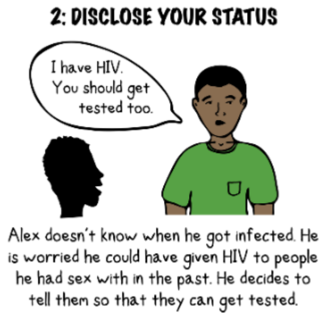

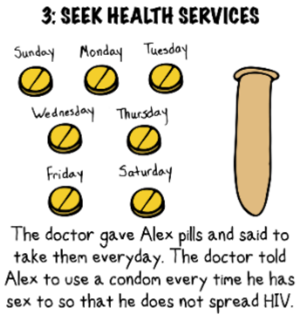

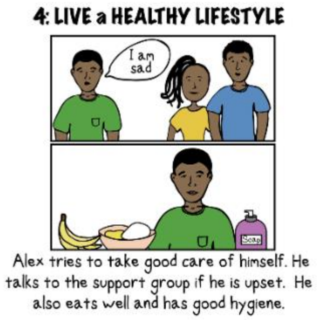

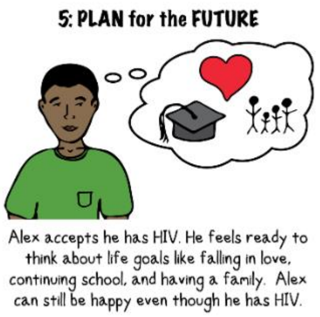

❑ **Step 3:**

Above the photo, where it reads, "Say something about this post" type the following message: "This story is about Alex and how he practices positive prevention 👌."

❑ **Step 4:**

Select "post" to post the photos and message.

DAY 6: DISCUSSION

❑ **Step 1:**

Sign into the Facebook Group

❑ **Step 2:**

Under the group title, next to your photo, under the text that reads "write something," type the following message: "What are you doing in your life to practice positive prevention?"

Note to Facilitator

As with other sessions, if you notice that certain group members have not yet participated this week, you might want to reach out to them privately to ask if everything is okay and to offer any support or advice they might need.

DAY 7: NO CONTENT TO POST

Monitor comments from participants. Encourage conversation by responding to participant posts and asking follow-up questions.

DAY 8: SOCIAL ACTIVITY

❑ **Step 1:**

Sign into the Facebook Group.

❑ Step 2:

Under the group title, next to your photo, under the text that reads “write something,” select the photo icon. Upload “Image #116” from your photo album.

A	B	C	D	E	F	G	H	I	J	K	L	M	N	O	P	Q	R	S
1	2	3	4	5	6	7	8	9	10	11	12	13	14	15	16	17	18	19
T	U	V	W	X	Y	Z												
20	21	22	23	24	25	26												

1	14	25	20	8	9	14	7
A					I		

9	19
I	

16	15	19	19	9	2	12	5
	O			I			E

❑ Step 3:

Above the photo, where it reads, “Say something about this post” type the following message: “Can you solve this puzzle? 🤔”

❑ Step 4:

Select “post” to post the photos and message.

Note to Facilitator

Each letter in this puzzle corresponds to a number as indicated in the first table. Using this table as a key, participants should try to “decode” the message below. The coded message is “Anything is possible.” If participants are stuck, feel free to give clues!

DAY 9: POLL

❑ Step 1:

Sign into the Facebook Group.

❑ Step 2:

Under the group title, next to your photo, under the text that reads “write something,”
select the poll icon.

❑ Step 3:

- Where you see the words “Ask something…” type the following: “Male and female condoms are the only method that can prevent pregnancy, STIs, and HIV.”
- Then where you see “+ Add a poll option…” type “True.” Click “Done.”
- Another “+ Add a poll option…” will appear. Type: “False.” Click “Done.”
- Select “Post” in the upper right corner.

DAY 10: POLL ANSWER

☐ Step 1:

Sign into the Facebook Group

☐ Step 2:

Under the poll you created yesterday, on the bottom right hand slide, click on the word "comment."

☐ Step 3:

Where you see the words "Write a comment…" type the following: "💯 the correct answer to this poll is true. Male and female condoms are the only method that prevents both pregnancy and STIs, including HIV. For condoms to work, you must use them correctly every time you have sex." Click the arrow icon to post your comment.

DAY 11: KEY MESSAGES

☐ Step 1:

Sign into the Facebook Group.

☐ Step 2:

Under the group title, next to your photo, under the text that reads "write something," select the photo icon. Upload images #117-130 from your photo album. Make sure that the "+Album" option is selected.

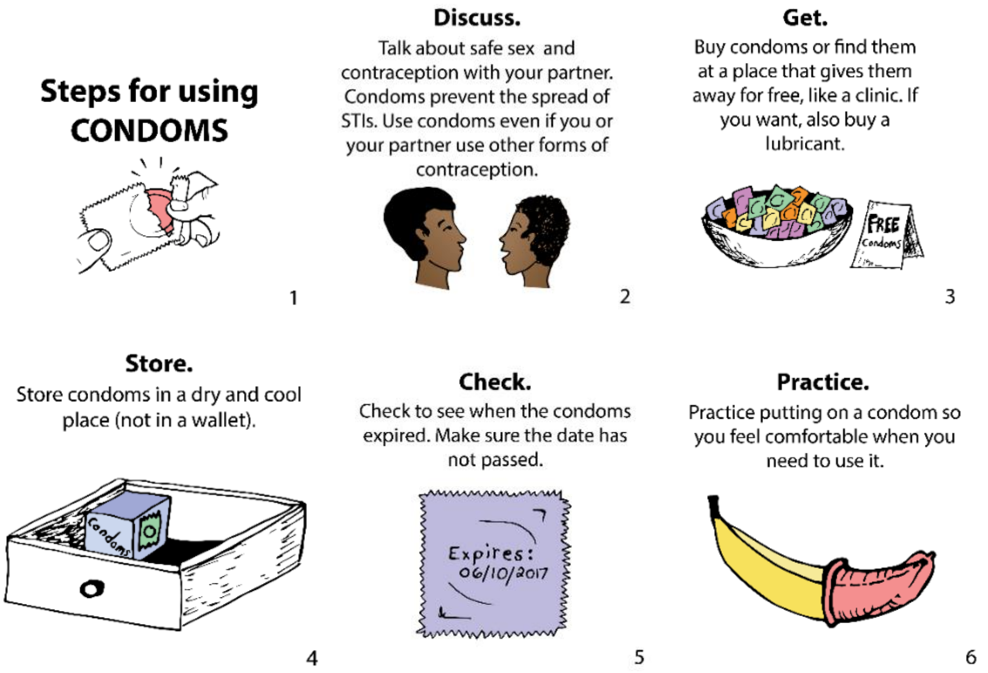

Lubricate.

Your body can make this naturally, you can buy a lubricant, or you can use saliva (spit). Never use one that is oil-based because it might cause the condom to tear.

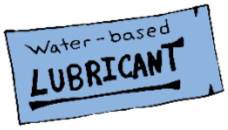

7

Open.

Try not to tear the condom when you open it. Fingernails, using your teeth, or rough handling can damage it.

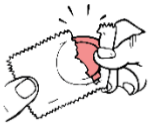

8

Squeeze.

When the penis is erect, squeeze the tip and place the condom on the head of the penis.

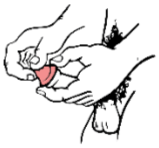

9

Unroll.

Hold the tip of the condom and unroll it until the penis is covered.

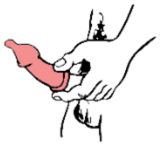

10

Broken condom?

If the condom breaks, pull out immediately.

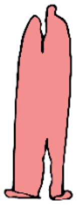

11

Pull out.

After ejaculation, when the penis is still erect, hold the end of the condom at the base of the penis.

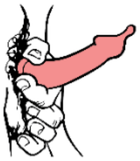

12

Remove.

Gently pull the penis out of the condom. Hold the end of the condom so you do not spill.

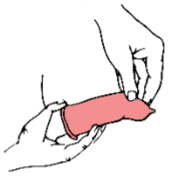

13

Throw away.

Tie up the condom or roll it in toilet paper and throw it away.

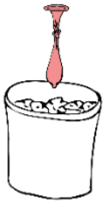

14

❑ **Step 3:**

Above the photo, where it reads, “Say something about this post” type the following message: “These photos show the steps for using condoms correctly.”

❑ **Step 4:**

Select “post” to post the photos and message.

DAY 12: DISCUSSION

❑ **Step 1:**

Sign into the Facebook Group.

❑ **Step 2:**

Under the group title, next to your photo, under the text that reads “write something,” type the following message: “Do you have any questions about positive prevention?”

❑ **Step 3:**

Click on the word "Post" in the upper right corner of the screen to post the message.

Note to Facilitator

As with other sessions, if you notice that certain group members have not yet participated this week, you might want to reach out to them privately to ask if everything is okay and to offer any support or advice they might need.

DAY 13: NO CONTENT TO POST

Monitor comments from participants. Encourage conversation by responding to participant posts and asking follow-up questions.

DAY 14: WRAP-UP

❑ **Step 1:**

Sign into the Facebook Group.

❑ **Step 2:**

Under the group title, next to your photo, under the text that reads "write something," type the following message: "We covered a lot in this session. Please let me know if you have any questions, need any clarification, or would like help finding a health care provider."

Note to Facilitator

As with other sessions, if you notice that certain group members have not yet participated this week, you might want to reach out to them privately to ask if everything is okay and to offer any support or advice they might need

PREPARE TO BEGIN SESSION 8

SESSION 8:
STIGMA, DISCRIMINATION, AND RIGHTS

Background for the Facilitator
Review the following information before leading this session.

Stigma means viewing someone negatively because of something that is different about the person; for example, they are living with HIV, they have a disability, or they are very poor. *Discrimination* means excluding people from opportunities that are available to other people. The stigma and discrimination associated with HIV can be destructive. The denial of basic rights—such as access to health care and treatment, opportunities for employment and housing—not only results in suffering and loss of dignity but can further contribute to the spread of the infection. For example, people might not seek HIV counseling, testing, treatment and support because having HIV could lead to rejection by family, friends or community, job loss or other negative consequences. That is why information and support are important for YLHIV. Young people need to know their rights in terms of employment, welfare, education and family life, and they need clear information about health care and treatment. YLHIV can be even more vulnerable to stigma and discrimination than others. Young people who acquired HIV through sex or injecting drug use might be more likely to be blamed for their status than someone who was born HIV positive.

This session focuses on stigma, discrimination, and rights.

Session Goals:

In this session, participants will:

- Learn about their rights regarding care and treatment, school, work, and life.
- Learn the meaning of stigma, and discrimination.

DAY 1: AT-A-GLANCE

❑ **Step 1:**

Sign into the Facebook Group.

❑ **Step 2:**

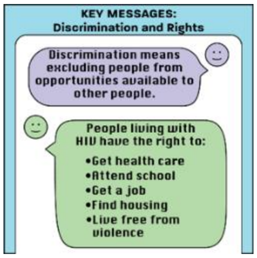

Under the group title, next to your photo, under the text that reads "write something,"
select the photo icon. Upload image #131 from your photo album. Click "done."

❑ **Step 3:**

Above the photo, where it reads, "Say something about this post" type the following message: *"During this next session we are going to learn about stigma, discrimination, and rights."*

❑ **Step 4:**

Select "post" to post the photo and message.

DAY 2: WORD OF THE WEEK

❑ **Step 1:**

Sign into the Facebook Group.

❑ **Step 2:**

Under the group title, next to your photo, under the text that reads "write something," select the photo icon. Upload Image #132 from your photo album. Click "done."

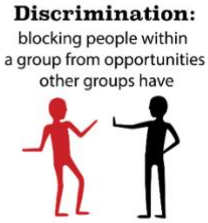

❑ **Step 3:**

Above the photo, where it reads, "Say something about this post" type the following message: "Check out this week's word of the week 👍."

❑ **Step 4:**

Select "post" to post the photo and message.

DAY 3: KEY MESSAGES

❑ Step 1:

Sign into the Facebook Group.

❑ Step 2:

Under the group title, next to your photo, under the text that reads "write something," select the photo icon. Upload images #133-138 from your photo album. Make sure that the "+Album" option is selected.

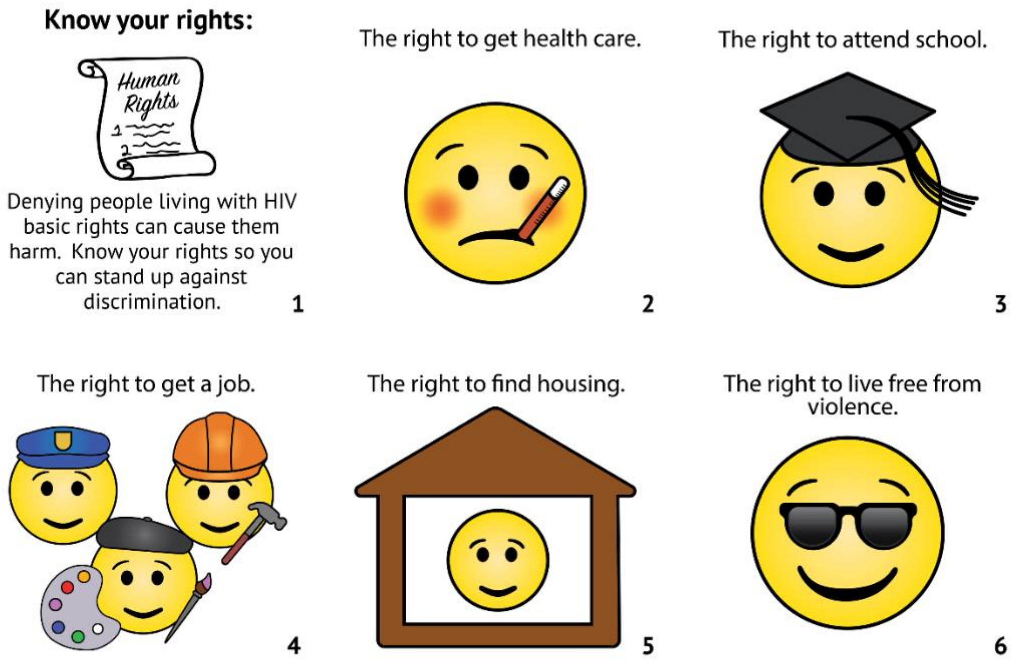

❑ Step 3:

Above the photo, where it reads, "Say something about this post" type the following message: "People living with HIV have the same rights as everyone else. These are some examples of rights."

❑ Step 4:

Select "post" to post the photos and message.

DAY 4: DISCUSSION

❑ Step 1:

Sign into the Facebook Group.

❑ **Step 2:**

Under the group title, next to your photo, under the text that reads "write something," type the following message: "What questions do you have about your rights and how they are protected?"

Note to Facilitator

As with other sessions, if you notice that certain group members have not yet participated this week, you might want to reach out to them privately to ask if everything is okay and to offer any support or advice they might need.

DAY 5: NO CONTENT TO POST

Monitor comments from participants. Encourage conversation by responding to participant posts and asking follow-up questions.

DAY 6: POLL

❑ **Step 1:**

Sign into the Facebook Group.

❑ **Step 2:**

Under the group title, next to your photo, under the text that reads "write something," select the poll icon.

❑ **Step 3:**

- Where you see the words "Ask something…" type the following: "Stigma means viewing someone negatively because of something that is different about them☹." Then where you see "+ Add a poll option…" type "True." Click "Done."
- Another "+ Add a poll option…" will appear. Type: "False." Click "Done."
- Select "Post" in the upper right corner.

DAY 7: POLL ANSWER

❑ **Step 1:**

Sign into the Facebook Group.

❑ **Step 2:**

Under the poll you created yesterday, on the bottom right hand side, click on the word "comment."

❑ Step 3:

Where you see the words "Write a comment…" type the following: "Stigma means not valuing a person and viewing them negatively because of something that is different about them. Stigma is not ok, you have the right to be treated the same as anyone else." Click the arrow icon to post your comment.

DAY 8: SOCIAL ACTIVITY

❑ Step 1:

Sign into the Facebook Group.

❑ Step 2:

Under the group title, next to your photo, under the text that reads "write something," type the following message: "It is Selfie day! Post a laughing selfie!"

Note to Facilitator

If you choose to post your own "Laughing selfie," select the photo icon, upload your selfie and type the message above in the "say something about this post" box.

DAY 9: KEY MESSAGE

❑ Step 1:

Sign into the Facebook Group.

❑ Step 2:

Under the group title, next to your photo, under the text that reads "write something," select the photo icon. Upload images #139-145 from your photo album. Make sure that the "+Album" option is selected.

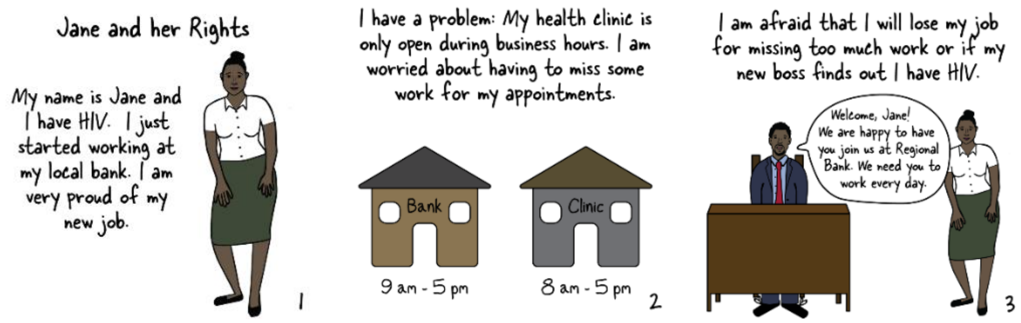

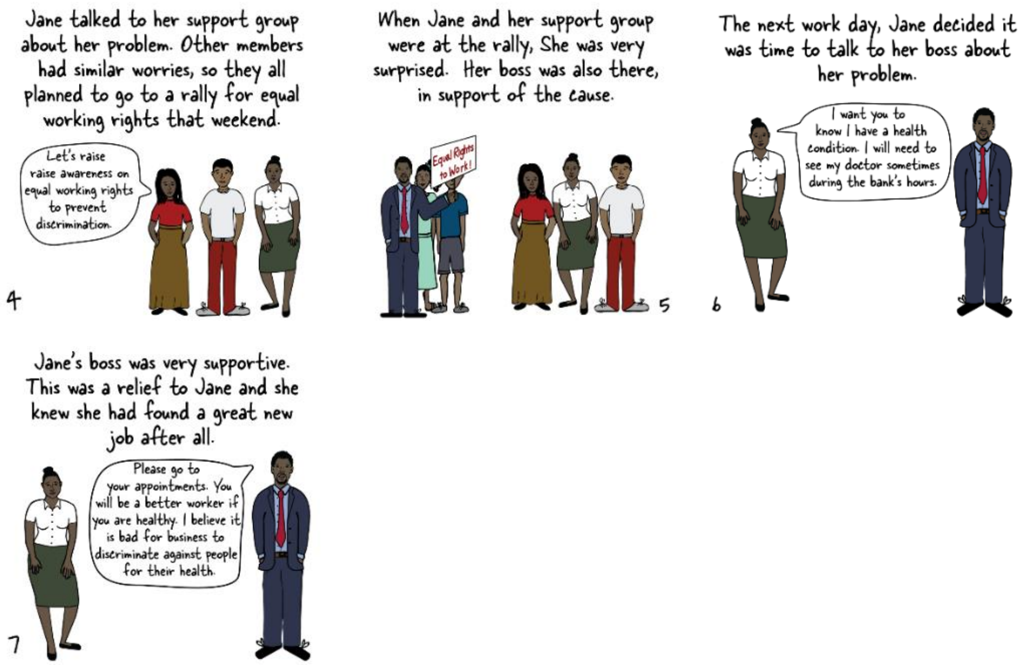

❑ **Step 3:**

Above the photo, where it reads, "Say something about this post" type the following message: "Check out this story of Jane and her rights."

❑ **Step 4:**

Select "post" to post the photos and message.

DAY 10: DISCUSSION

❑ **Step 1:**

Sign into the Facebook Group.

❑ **Step 2:**

Under the group title, next to your photo, under the text that reads "write something," type the following message: "If you are comfortable doing so, share a time when you felt discriminated against or worried that you might face discrimination. How did it make you feel? What did you do?"

❑ **Step 3:**

Click on the word "Post" in the upper right corner of the screen to post the message.

Note to Facilitator

I Thank participants who share for sharing their experiences and monitor the discussion closely to ensure that all participants are responding in a way that is supportive to one another. As with other sessions, if you notice that certain group members have not yet participated this week, you might want to reach out to them privately to ask if everything is okay and to offer any support or advice they might need.

DAY 11: NO CONTENT TO POST

Monitor comments from participants. Encourage conversation by responding to participant posts and asking follow-up questions.

DAY12: RESOURCE

❑ **Step 1:**

Sign into the Facebook Group.

❑ **Step 2:**

Under the group title, next to your photo, under the text that reads "write something," type the following: https://www.greaterthan.org/stories/ An image that says, "Greater Than," should appear.

❑ **Step 3:**

Next to the website link, type the following message: "Check out this website for real stories about people living with HIV in the US."

❑ **Step 4:**

Click on the word "Post" in the upper right corner of the screen to post the website and message.

DAY 13: SOCIAL ACTIVITY

❑ **Step 1:**

Sign into the Facebook Group.

❑ **Step 2:**

Under the group title, next to your photo, under the text that reads "write something," type the following message: "Share your favorite inspirational quote."

Note to Facilitator

In addition to this message, you may choose to share your own favorite inspirational quote.

DAY 14: WRAP-UP

❑ Step 1:

Sign into the Facebook Group.

❑ Step 2:

Under the group title, next to your photo, under the text that reads "write something," type the following message: "Thank you for sharing this week! Facing stigma and discrimination can be hard to deal with. I am here to support you and you can support each other!"

Note to Facilitator

As with other sessions, if you notice that certain group members have not yet participated this week, you might want to reach out to them privately to ask if everything is okay and to offer any support or advice they might need.

PREPARE TO BEGIN SESSION 9.

SESSION 9: VIOLENCE

Background for the Facilitator
Review the following information before leading this session.

Violence and HIV infection are linked in several ways:

- Violence can lead to HIV, such as when a young person is infected with HIV through an act of sexual violence.
- Violence in a person's past can cause emotional trauma that may lead young people to engage in high-risk behaviors. This trauma may also lower a young person's self-esteem make him or her less likely to seek HIV treatment if it is needed.
- YLHIV may be targeted with violence because of their HIV status.

This session will focus on the increased threat of violence that YLHIV may experience because of their HIV status. Violence can be perpetrated by oneself (such as suicide) or other individuals (such as bullying, physical violence and rape). In addition to facing violence in their homes, schools, or communities, young people using social media face the risk of cyber bullying. Cyber bullying is bullying that takes place online, and it can have serious consequences.

Because YLHIV are vulnerable to violence, it is possible that support group participants might disclose experiences of violence during this session. Facilitators must be prepared to respond to those who talk about being victimized. Review the training materials on responding to violence and ensure that you have an up-to-date list of local referral resources. Nigeria suicide prevention hotline information is available at this website: https://sspinitiative1.wixsite.com/sspi

Your role as an influential and trusted adult is to help young people do the following:

- Identify violence and recognize their right to live without it
- Understand that they are not at fault and should not blame themselves
- Find professional help if needed

In this session, we describe violence between individuals. Remind the group that violence between individuals is a sensitive and complicated subject and that what people say during this or any session should not be shared outside of the group. Also, remind participants that they can talk with you directly if they do not want to share with the group. Participants are here to help and not judge one another.

Session Goals:

In this session, participants will:

- Learn the definition of violence and about their right to live without it
- Learn the different forms of violence
- Understand that they are not at fault and should not blame themselves

DAY 1: AT-A-GLANCE

❑ Step 1:

Sign into the Facebook Group.

❑ Step 2:

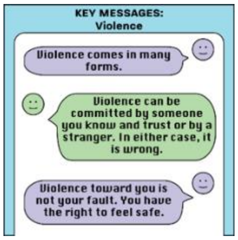

Under the group title, next to your photo, under the text that reads "write something," select the photo icon. Upload "Image #146" from your photo album. Click "done"

❑ Step 3:

Above the photo, where it reads, "Say something about this post" type the following message: *"During this next session we are going to talk about violence."*

Violence:
an action or behaviour meant to cause harm

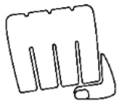

❑ Step 4:

Select "post" to post the photo and message.

DAY 2: WORD OF THE WEEK

❑ Step 1:

Sign into the Facebook Group.

❑ Step 2:

Under the group title, next to your photo, under the text that reads "write something," select the photo icon. Upload "Image #147" from your photo album. Click "done"

❑ Step 3:

Above the photo, where it reads, "Say something about this post" type the following message: "Check out this week's word of the week."

❑ Step 4:

Select "post" to post the photo and message.

DAY 3: KEY MESSAGES

❑ Step 1:

Sign into the Facebook Group

❑ Step 2:

Under the group title, next to your photo, under the text that reads "write something," select the photo icon. Upload images #148-158 from your photo album. Make sure that the "+Album" option is selected.

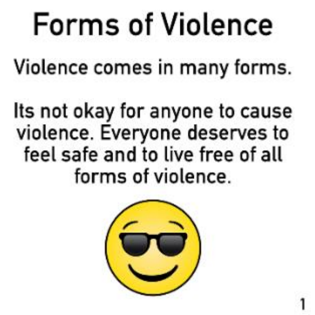

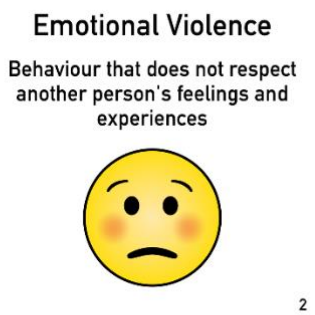

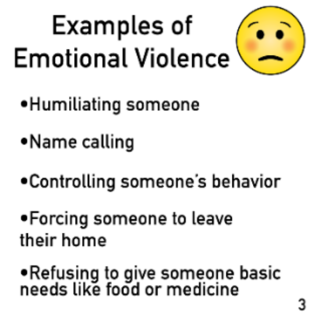

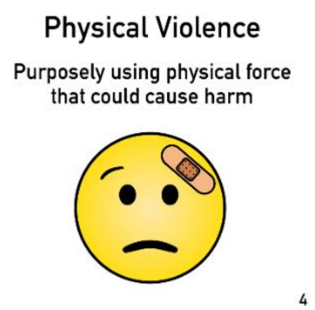

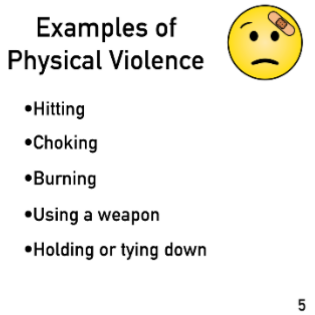

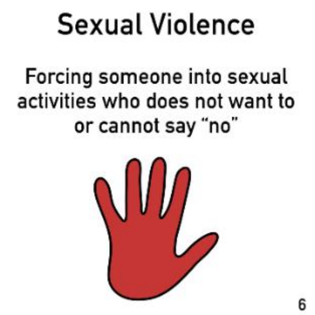

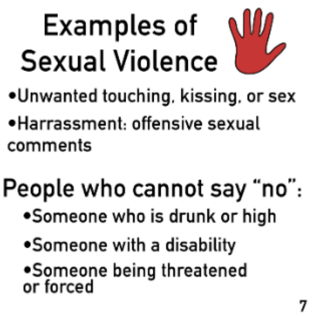

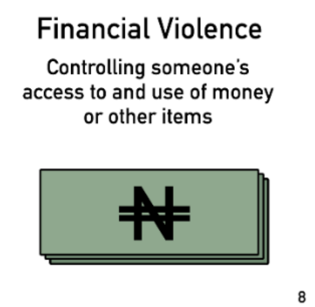

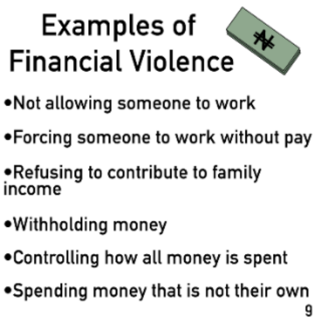

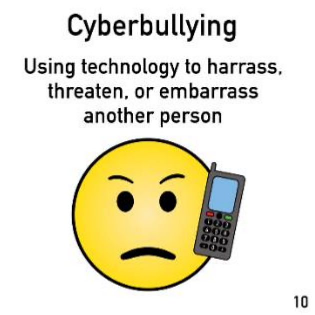

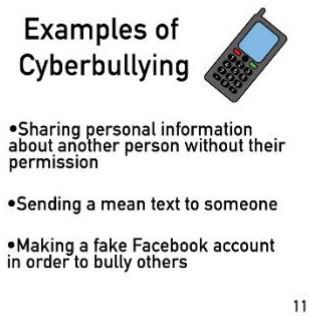

❑ **Step 3:**

Above the photo, where it reads, "Say something about this post" type the following message: "Violence comes in many forms…everyone has the right to live a life free of violence."

❑ **Step 4:**

Select "post" to post the photos and message.

DAY 4: DISCUSSION

❑ **Step 1:**

Sign into the Facebook Group.

❑ **Step 2:**

Under the group title, next to your photo, under the text that reads "write something," type the following message: "Sometimes, people blame themselves if they experience violence—but violence is never the victim's fault. What could you tell a friend who experienced violence to help them understand that was not their fault?"

Note to Facilitator

As with other sessions, if you notice that certain group members have not yet participated this week, you might want to reach out to them privately to ask if everything is okay and to offer any support or advice they might need.

DAY 5: NO CONTENT TO POST

Monitor comments from participants. Encourage conversation by responding to participant posts and asking follow-up questions.

DAY 6: POLL

❑ **Step 1:**

Sign into the Facebook Group

❑ **Step 2:**

Under the group title, next to your photo, under the text that reads "write something," select the poll icon.

- [ ] **Step 3:**

- Where you see the words "Ask something…" type the following:
  "People with HIV are targeted with violence because they deserve it."
  Then where you see "+ Add a poll option…" type "True." Click "Done."
- Another "+ Add a poll option…" will appear. Type: "False." Click "Done."
- Select "Post" in the upper right corner.

DAY 7: POLL ANSWER

- [ ] **Step 1:**

Sign into the Facebook Group.

- [ ] **Step 2:**

Under the poll you created yesterday, on the bottom right hand slide, click on the word "comment."

- [ ] **Step 3:**

Where you see the words "Write a comment…" type the following:
"This is FALSE. People living with HIV may be targeted with violence for many reasons, but they DO NOT deserve violence. No one deserves to experience violence." Click the arrow icon to post your comment.

DAY 8: SOCIAL ACTIVITY

- [ ] **Step 1:**

Sign into the Facebook Group.

- [ ] **Step 2:**

Under the group title, next to your photo, under the text that reads "write something," select the photo icon. Upload "Image #138" from your photo album.

A	B	C	D	E	F	G	H	I	J	K	L	M	N	O	P	Q	R	S
1	2	3	4	5	6	7	8	9	10	11	12	13	14	15	16	17	18	19
T	U	V	W	X	Y	Z												
20	21	22	23	24	25	26												

25	15	21
Y		

1	18	5
	R	

19	20	18	15	14	7
		R			G

- [ ] **Step 3:**

Above the photo, where it reads, "Say something about this post" type the following message: "Can you solve this puzzle? 🤔"

- [ ] **Step 4:**

Select "post" to post the photos and message.

Note to Facilitator

Each letter in this puzzle corresponds to a number as indicated in the first table. Using this table as a key, participants should try to "decode" the message below. The coded message is "You are strong." If participants are stuck, feel free to give clues!

DAY 9: POLL

❑ Step 1:

Sign into the Facebook Group.

❑ Step 2:

Under the group title, next to your photo, under the text that reads "write something," select the poll icon.

❑ Step 3:

- Where you see the words "Ask something…" type the following: "Emotional violence, such as teasing someone, is not really harmful." Then where you see "+ Add a poll option…" type "True." Click "Done."
- Another "+ Add a poll option…" will appear. Type: "False." Click "Done."
- Select "Post" in the upper right corner.

DAY 10: POLL ANSWER

❑ Step 1:

Sign into the Facebook Group.

❑ Step 2:

Under the poll you created yesterday, on the bottom right hand side, click on the word "comment."

❑ Step 3:

Where you see the words "Write a comment…" type the following: "The correct response is False. Emotional violence can be just as harmful as any other form of violence." Click the arrow icon to post your comment.

DAY 11: DISCUSSION

❑ Step 1:

Sign into the Facebook Group.

❑ **Step 2:**

Under the group title, next to your photo, under the text that reads "write something," type the following message: "What can you do if someone you know is experiencing violence?"

❑ **Step 3:**

Click on the word "Post" in the upper right corner of the screen to post the website and message.

Note to Facilitator

Thank participants who share their experiences and monitor the discussion closely to ensure that all participants are responding in a way that is supportive to one another. As with other sessions, if you notice that certain group members have not yet participated this week, you might want to reach out to them privately to ask if everything is okay and to offer any support or advice they might need.

DAY 12: NO CONTENT TO POST

Monitor comments from participants. Encourage conversation by responding to participant posts and asking follow-up questions.

DAY 13: SOCIAL ACTIVITY

❑ **Step 1:**

Sign into the Facebook Group.

❑ **Step 2:**

Under the group title, next to your photo, under the text that reads "write something," type the following message: "It is Selfie day! Post a dancing selfie! 💃"

Note to Facilitator

If you choose to post your own "Dancing selfie," select the photo icon, upload your selfie and type the message above in the "say something about this post" box.

DAY 14: WRAP-UP

❑ **Step 1:**

Sign into the Facebook Group.

❑ Step 2:

Under the group title, next to your photo, under the text that reads “write something,” type the following message: “Thank you for sharing this week! This was a difficult topic. If you have experienced violence, know you are not alone. I am here to support you, we can all support each other.”

Note to Facilitator

As with other sessions, if you notice that certain group members have not yet participated this week, you might want to reach out to them privately to ask if everything is okay and to offer any support or advice they might need.

PREPARE TO BEGIN SESSION 10

SESSION 10: COMMUNICATION AND PROBLEM SOLVING

Background for the Facilitator

Review the following information before leading this session.

Everyone needs good communication and problem-solving skills to meet the demands of everyday life. These demands are different for YLHIV, who must be able to speak about living with HIV with their health care providers, caretakers, friends, family, sex partners and people in their community. Some examples of effective communication skills include *speaking to be understood*, *listening to understand* and *being assertive*. Problem-solving is about dealing with problems in a logical, effective way. All of these skills play a role in developing and maintaining healthy family, community and romantic relationships.

Session Goals:

In this session, participants will:

- Discuss how communication affects their relationship with family, caregivers, health care providers and others in their communities.
- Learn how to be good communicators and why this is especially important for YLHIV.

DAY 1: AT-A-GLANCE

❑ **Step 1:**

Sign into the Facebook Group

❑ **Step 2:**

Under the group title, next to your photo, under the text that reads "write something," select the photo icon. Upload "Image #160 from your photo album. Click "done."

❑ **Step 3:**

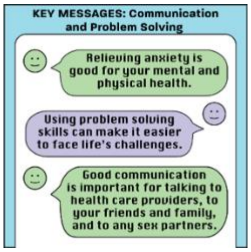

Above the photo, where it reads, "Say something about this post" type the following message: *"This session will cover communication and problem solving."*

❑ **Step 4:**

Select "post" to post the photo and message.

DAY 2: WORD OF THE WEEK

❑ **Step 1:**

Sign into the Facebook Group

❑ **Step 2:**

Under the group title, next to your photo, under the text that reads "write something," select the photo icon. Upload "Image #161" from your photo album. Click "done"

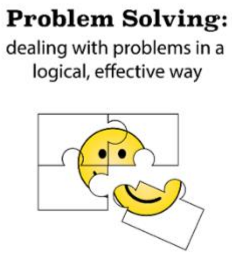

❑ **Step 3:**

Above the photo, where it reads, "Say something about this post" type the following message: "Check out this week's word of the week 😎."

❑ **Step 4:**

Select "post" to post the photo and message.

DAY 3: DISCUSSION

❑ Step 1:

Sign into the Facebook Group.

❑ Step 2:

Under the group title, next to your photo, under the text that reads "write something," type the following message: "Think of a time when you had to solve a problem. What was the problem? What were the steps you took to solve the problem? What worked? What would you do differently?"

❑ Step 3:

Click on the word "Post" in the upper right corner of the screen to post the website and message.

Note to Facilitator

Thank participants who share for sharing their experiences and monitor the discussion closely to ensure that all participants are responding in a way that is supportive to one another. As with other sessions, if you notice that certain group members have not yet participated this week, you might want to reach out to them privately to ask if everything is okay and to offer any support or advice they might need.

DAY 4: KEY MESSAGES

❑ Step 1:

Sign into the Facebook Group.

❑ Step 2:

Under the group title, next to your photo, under the text that reads "write something," select the photo icon. Upload images #162-166 from your photo album. Make sure that the "+Album" option is selected.

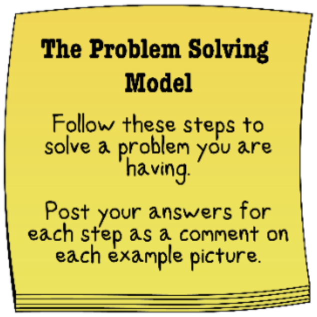

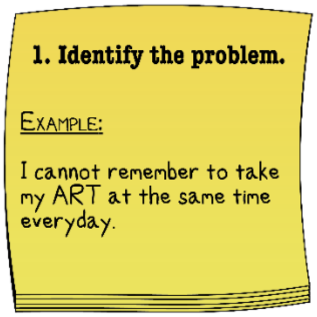

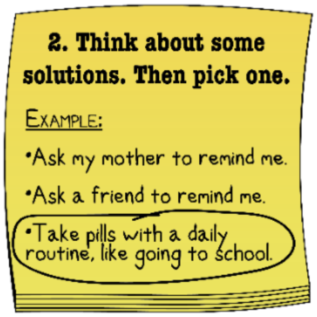

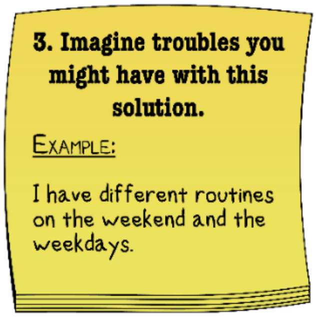

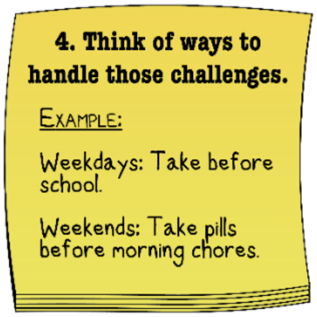

❑ Step 3:

Above the photo, where it reads, "Say something about this post" type the following message: "Check out these problem-solving steps."

❑ Step 4:

Select "post" to post the photos and message.

DAY 5: DISCUSSION

❑ Step 1:

Sign into the Facebook Group.

❑ Step 2:

Under the group title, next to your photo, under the text that reads "write something," type the following message: "Think of a person you know who is a good communicator." What makes that person a good communicator?"

Note to Facilitator

As with other sessions, if you notice that certain group members have not yet participated this week, you might want to reach out to them privately to ask if everything is okay and to offer any support or advice they might need.

DAY 6: NO CONTENT TO POST

Monitor comments from participants. Encourage conversation by responding to participant posts and asking follow-up questions.

DAY 7: SOCIAL ACTIVITY

❑ Step 1:

Sign into the Facebook Group.

❑ Step 2:

Under the group title, next to your photo, under the text that reads "write something," the following message: "Fill in the blank. Something that makes me happy ☺ is ___________"

❑ Step 3:

Select "post" to post the message.

DAY 8: GRACE'S STORY

❑ Step 1:

Sign into the Facebook Group.

❑ Step 2:

Under the group title, next to your photo, under the text that reads "write something," select the photo icon. Upload images #167-172 from your photo album. Make sure that the "+Album" option is selected.

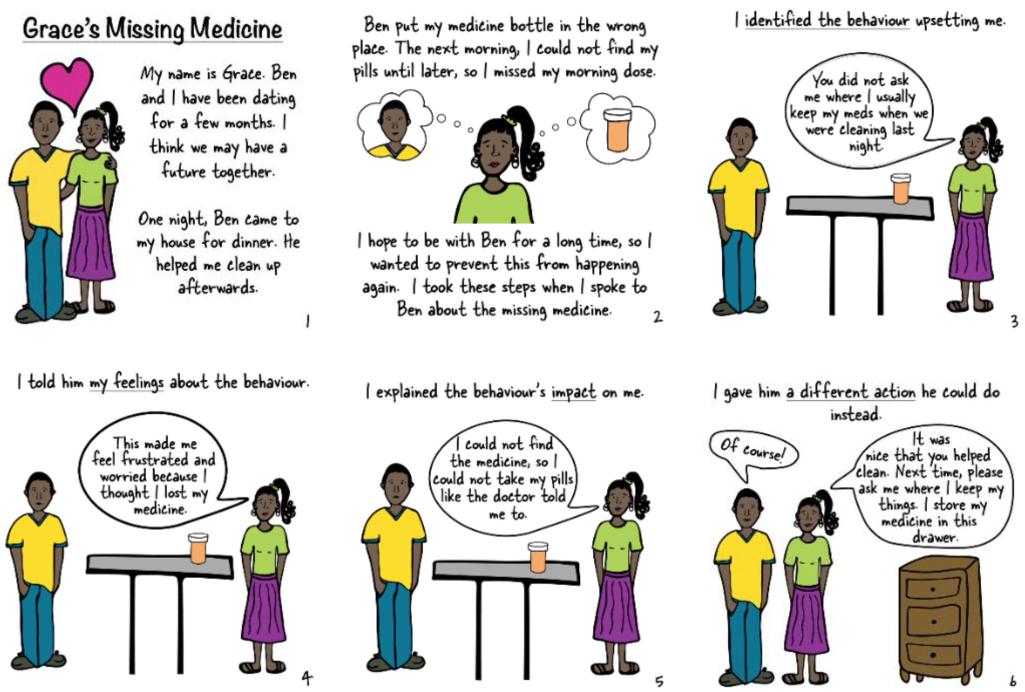

❑ Step 3:

Above the photo, where it reads, "Say something about this post" type the following message: "This story is about how Grace deals with conflict."

❑ Step 4:

Select "post" to post the photos and message.

DAY 9: DISCUSSION

❑ **Step 1:**

Sign into the Facebook Group.

❑ **Step 2:**

Under the group title, next to your photo, under the text that reads "write something," type the following message: "Think of a time you had a conflict or problem related to your HIV status. How did you handle it?"

Note to Facilitator

As with other sessions, if you notice that certain group members have not yet participated this week, you might want to reach out to them privately to ask if everything is okay and to offer any support or advice they might need.

DAY 10: NO CONTENT TO POST

Monitor comments from participants. Encourage conversation by responding to participant posts and asking follow-up questions.

DAY 11: POLL

❑ **Step 1:**

Sign into the Facebook Group.

❑ **Step 2:**

Under the group title, next to your photo, under the text that reads "write something," select the poll icon.

❑ **Step 3:**

- Where you see the words "Ask something…" type the following: "Which of these is *NOT* a problem-solving step?" Then where you see "+ Add a poll option…" type "Identify the problem." Click "Done."
- Another "+ Add a poll option…" will appear. Type: "Identify solutions." Click "Done."
- Another "+ Add a poll option…" will appear. Type: "Evaluate solutions and choose one." Click "Done."
- Another "+ Add a poll option…" will appear. Type: "Identify possible challenges to the solution." Click "Done."
- Another "+ Add a poll option…" will appear. Type: "Deal with challenges as they arise." Click "Done."

- Another “+ Add a poll option…” will appear. Type: “All of these.” Click “Done.” Select “Post” in the upper right corner.

DAY 13: POLL ANSWER

❑ Step 1:

Sign into the Facebook Group.

❑ Step 2:

Under the poll you created yesterday, on the bottom right hand side, click on the word “comment.”

❑ Step 3:

Where you see the words “Write a comment…” type the following: “The correct answer to this poll is “Deal with challenges as they arise.” Once you identify possible challenges, it is important to also identify ways to handle these challenges *before* they arise.” Click the arrow icon to post your comment.

DAY 14: WRAP-UP

❑ Step 1:

Sign into the Facebook Group.

❑ Step 2:

Under the group title, next to your photo, under the text that reads “write something,” type the following message: “Thank you for participating this week. Remember it takes practice to be assertive and be a good problem solver.”

Note to Facilitator

As with other sessions, if you notice that certain group members have not yet participated this week, you might want to reach out to them privately to ask if everything is okay and to offer any support or advice they might need.

PREPARE TO BEGIN SESSION 11.

SESSION 11:
PLANNING FOR YOUR FUTURE

Background for the Facilitator
Review the following information before leading this session.

As YLHIV continue to mature, they will be expected to play a larger role not only in managing their own care and treatment, but in contributing to their households and the community at large.

A general lack of means or finances can play a large role in increasing the vulnerability of YLHIV to STIs, pregnancy and transactional sex. However, with the security of a steady income, a good education, or a job or career, a YLHIV is more likely to:

- Have greater self-esteem and confidence
- Make plans for their future
- Wait to have sex until they are ready
- Stand up for themselves
- Delay having children until they are older

An essential component of care and treatment is psychosocial well-being. Planning for the future and having a sense of hope helps cultivate mental and spiritual health and gives YLHIV a sense of purpose and belonging.

DAY 1: AT-A-GLANCE

❑ Step 1:

Sign into the Facebook Group

❑ Step 2:

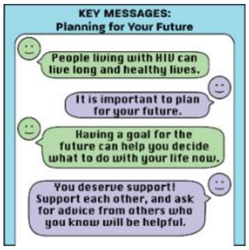

Under the group title, next to your photo, under the text that reads "write something," select the photo icon. Upload "Image #173" from your photo album. Click "done"

Step 3:

Above the photo, where it reads, "Say something about this post" type the following message: "This session is about planning for your future."

Step 4:

Select "post" to post the photo and message.

DAY 2: WORD OF THE WEEK

❑ Step 1:

Sign into the Facebook Group.

❑ Step 2:

Under the group title, next to your photo, under the text that reads "write something," select the photo icon. Upload "Image #174 from your photo album. Click "done"

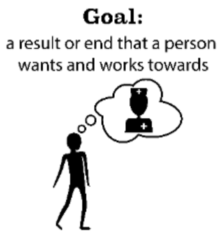

❑ Step 3:

Above the photo, where it reads, "Say something about this post" type the following message: "Check out this week's word of the week ."

❑ Step 4:

Select "post" to post the photo and message.

DAY 3: KEY MESSAGES

❑ Step 1:

Sign into the Facebook Group.

❑ Step 2:

Under the group title, next to your photo, under the text that reads "write something," select the photo icon. Upload images #175-181 from your photo album. Make sure that the "+Album" option is selected.

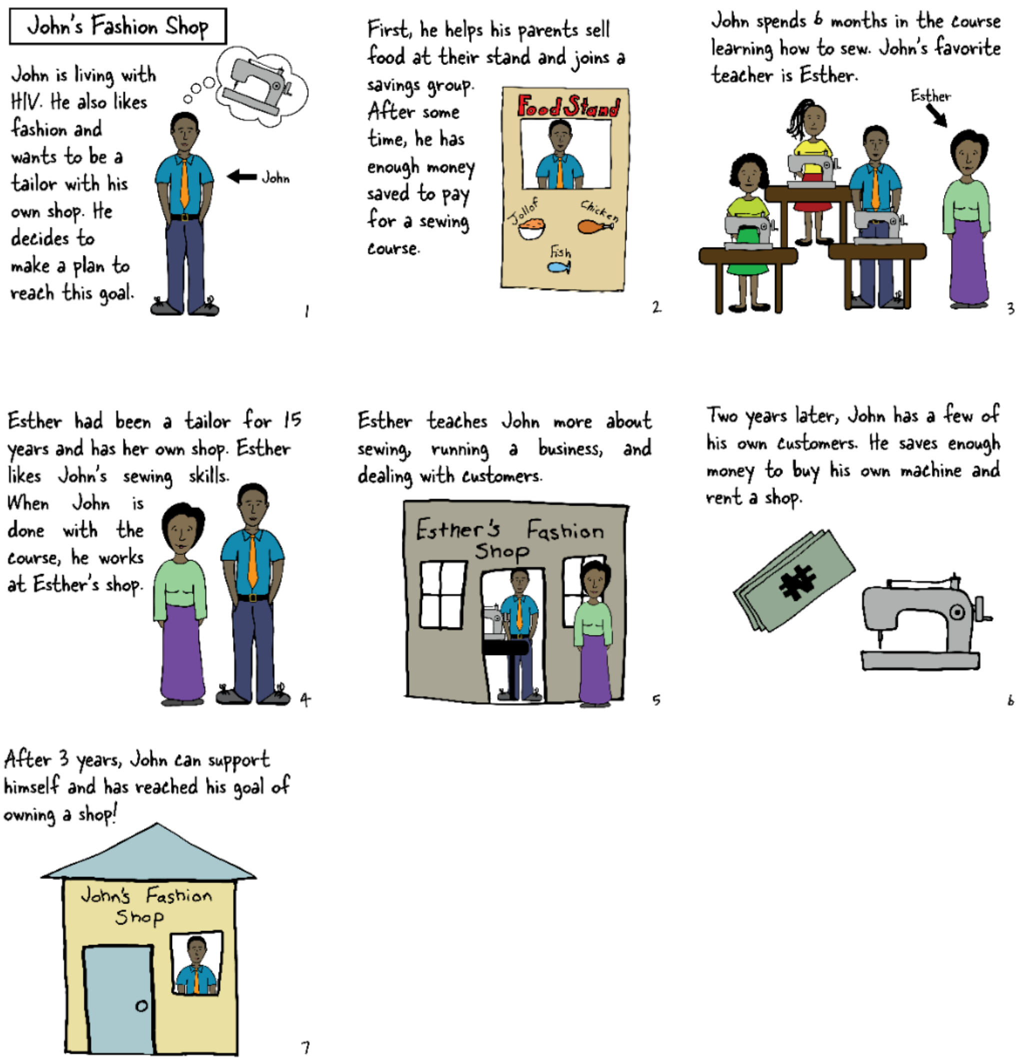

❑ Step 3:

Above the photo, where it reads, "Say something about this post" type the following message: "Check out how John achieved his goal of owning a fashion shop 💯."

❑ Step 4:

Select "post" to post the photos and message.

DAY 4: DISCUSSION

❑ Step 1:

Sign into the Facebook Group.

❑ Step 2:

Under the group title, next to your photo, under the text that reads "write something," type the following message: "What factors lead to John's success in achieving his goal?"

Note to Facilitator

As with other sessions, if you notice that certain group members have not yet participated this week, you might want to reach out to them privately to ask if everything is okay and to offer any support or advice they might need.

DAY 5: NO CONTENT TO POST

Monitor comments from participants. Encourage conversation by responding to participant posts and asking follow-up questions.

DAY 6: POLL

❑ Step 1:

Sign into the Facebook Group.

❑ Step 2:

Under the group title, next to your photo, under the text that reads "write something,"
select the poll icon.

❑ Step 3:

- Where you see the words "Ask something…" type the following:
  "It is important for people living with HIV to plan for the future."
  Then where you see "+ Add a poll option…" type "True." Click "Done."
- Another "+ Add a poll option…" will appear. Type: "False." Click "Done."
- Select "Post" in the upper right corner.

DAY 7: POLL ANSWER

❑ **Step 1:**

Sign into the Facebook Group.

❑ **Step 2:**

Under the poll you created yesterday, on the bottom right hand side, click on the word "comment."

❑ **Step 3:**

Where you see the words "Write a comment…" type the following: "The correct response is True. People living with HIV can live long and healthy lives, so it is important to plan for your future. Having a goal for the future can help you decide what to do with your life now."

DAY 8: SOCIAL ACTIVITY

❑ **Step 1:**

Sign into the Facebook Group.

Step 2:

Under the group title, next to your photo, under the text that reads "write something," type the following message: "It is Selfie day! Post a #lifegoals selfie!"

Note to Facilitator

If you choose to post your own "#lifegoals selfie," select the photo icon, upload your selfie and type the message above in the "say something about this post" box.

DAY 9: PATIENCE'S STORY

❑ **Step 1:**

Sign into the Facebook Group.

❑ **Step 2:**

Under the group title, next to your photo, under the text that reads "write something," select the photo icon. Upload images #182-187 from your photo album. Make sure that the "+Album" option is selected.

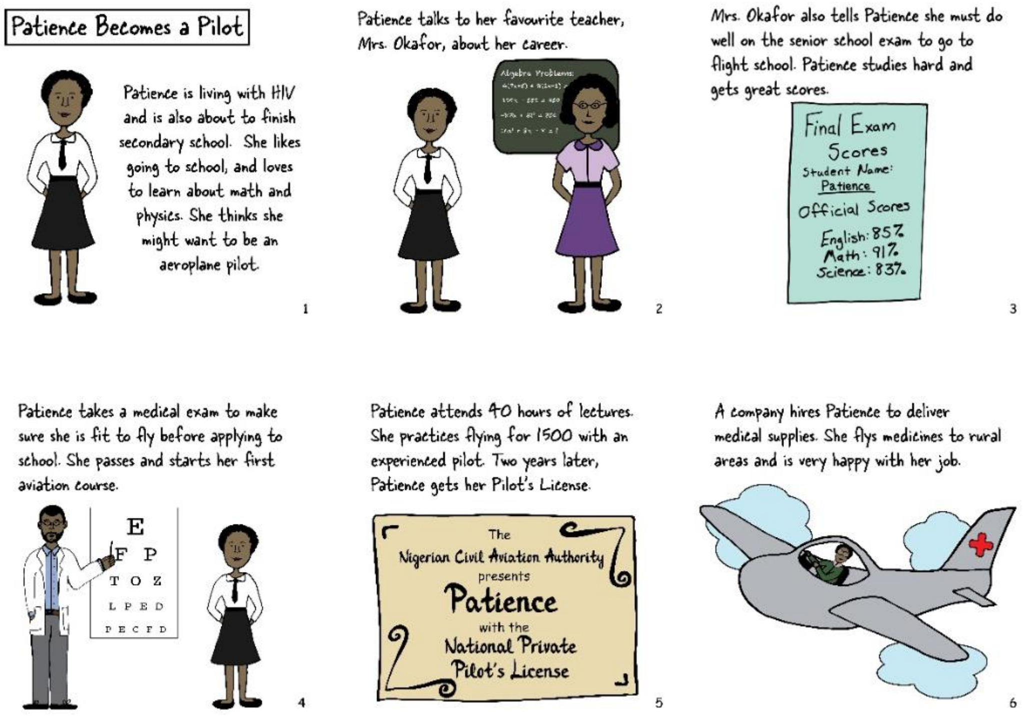

❑ Step 3:

Above the photo, where it reads, "Say something about this post" type the following message: "This is the story of how Patience became a pilot ."

❑ Step 4:

Select "post" to post the photos and message.

DAY 10: DISCUSSION

❑ Step 1:

Sign into the Facebook Group.

❑ Step 2:

Under the group title, next to your photo, under the text that reads "write something," type the following message: "What goals do you have for your future? "

Note to Facilitator

As with other sessions, if you notice that certain group members have not yet participated this week, you might want to reach out to them privately to ask if everything is okay and to offer any support or advice they might need.

DAY 11: MOST IMPORTANT LESSON/ SKILL SOCIAL ACTIVITY CLOSING INTENTION

❑ Step 1:

Sign into the Facebook Group.

❑ Step 2:

Under the group title, next to your photo, under the text that reads “write something,” type the following message: “What is the most important lesson or skill you have learned through our time together and commit to apply in your life? Share your intention by replying to this post 🐱 🏍.”

❑ Step 3:

Monitor comments from participants and provide responses to questions.

Note to Facilitator

As with other sessions, if you notice that certain group members have not yet participated this week, you might want to reach out to them privately to ask if everything is okay and to offer any support or advice they might need.

DAY 12: DISCUSSION

❑ Step 1:

Sign into the Facebook Group

❑ Step 2:

Under the group title, next to your photo, under the text that reads “write something,” type the following message: “Our time together is coming to a close. We need to decide as group if we will keep the group open informally. Please respond to this post with your personal preference. It is ok if some people choose to stay in the group, but others stop participating.”

Note to Facilitator

There are four possible options for the group at the close of this program:

1. The group remains intact. You remain the group administrator. In this case no action is needed.

2.. Some group members decide to leave the group, while others choose to continue to participate. As the administrator you must remove group members who no longer wish to participate.

3. The group remains intact, in part or in whole, but you no longer wish to be the administrator. In this case you must make another group member the administrator, and then remove yourself from the secret group.

4. No group members wish to participate beyond the program period. In this case, as the administrator you must close the group.

DAY 13: SOCIAL ACTIVITY—COMPLIMENT CIRCLE

❑ **Step 1:**

Sign into the Facebook Group

❑ **Step 2:**

Under the group title, next to your photo, under the text that reads "write something," type the following message: "As a final activity—lets show how much we appreciate each other. Share something kind about one or more of your groupmates."

Note to Facilitator

You may choose to post a compliment about each group member. Please ensure that each group member receives at least one compliment.

DAY 14: WRAP-UP

❑ **Step 1:**

Sign into the Facebook Group

❑ **Step 2:**

Under the group title, next to your photo, under the text that reads "write something," type the following message: "Today is our last day 😢. Thank you for all your hard work and participation! Even though we will no longer officially meet, you can always use this group for information and to support each other.

CITATIONS

1. Murray KR, Dulli LS, Ridgeway K, Dal Santo L, Darrow de Mora D, Olsen P, Silverstein H, McCarraher DR. Improving retention in HIV care among adolescents and adults in low- and middle-income countries: A systematic review of the literature. PLoS One. 2017 Sep 29;12(9):e0184879.

2. Ridgeway K, Dulli LS, Murray KR, Silverstein H, Dal Santo L, Olsen P, Darrow de Mora D, McCarraher DR. Interventions to improve antiretroviral therapy adherence among adolescents in low- and middle-income countries: A systematic review of the literature. PLoS One. 2018 Jan 2;13(1):e0189770.

3. Dulli L, Ridgeway K, Packer C, Plourde KF, Mumuni T, Idaboh T, Olumide A, Ojengbede O, McCarraher DR. An Online Support Group Intervention for Adolescents Living with HIV in Nigeria: A Pre-Post Test Study. JMIR Public Health Surveill. 2018 Nov 28;4(4):e12397.

4. FHI 360. Positive Connections: Leading Information and Support Groups for Adolescents Living with HIV. 2013. FHI 360: Durham, NC.

ANNEX:
REFERRAL CHART

Organizations or clinics that provide HIV-related services

Name of organization	Address	Telephone	Contact name	Email address
1				
2				
3				
4				

Organizations or clinics that provide contraceptive services

Name of organization	Address	Telephone	Contact name	Email address
1				
2				
3				
4				

Organizations or clinics that provide STI screening or treatment

Name of organization	Address	Telephone	Contact name	Email address
1				
2				
3				
4				

Organizations or clinics that provide care for victims of sexual violence

Name of organization	Address	Telephone	Contact name	Email address
1				
2				
3				
4				

Organizations or clinics that provide psychological or mental health counseling

Name of organization	Address	Telephone	Contact name	Email address
1				
2				
3				
4				

Organizations that work with adolescents (such as YWCA, YMCA, scouts, youth groups)

Name of organization	Address	Telephone	Contact name	Email address
1				
2				
3				
4				

Organizations that can provide information and training related to job skills, livelihoods or education

Name of organization	Address	Telephone	Contact name	Email address
1				
2				
3				
4				

Organizations that can work with victims of bullying or school violence

Name of organization	Address	Telephone	Contact name	Email address
1				
2				
3				
4				

Organizations or clinics that provide services for prevention of mother-to-child transmission of HIV

Name of organization	Address	Telephone	Contact name	Email address
1				
2				
3				
4				

Pharmacies or other locations that can provide information on ARVs (including information on storage, adherence and side effects)

Name of organization	Address	Telephone	Contact name	Email address
1				
2				
3				
4				

Organizations or clinics that provide nutrition counseling and help

Name of organization	Address	Telephone	Contact name	Email address
1				
2				
3				
4				

Organizations that have information related to the rights of PLHIV

Name of organization	Address	Telephone	Contact name	Email address
1				
2				
3				
4				

This annex was reprinted from FHI 360. Positive Connections: Leading Information and Support Groups for Adolescents Living with HIV. 2013. FHI 360: Durham, NC.

USAID/YouthPower Action

Social Media to Improve Art Retention and Treatment Outcomes
Among Youth Living with HIV in Nigeria (SMART) Connections Program Guide
